# Rational Design of Stapled Antimicrobial Peptides to Enhance Stability and *In Vivo* Potency against Polymicrobial Sepsis

**DOI:** 10.1128/spectrum.03853-22

**Published:** 2023-03-06

**Authors:** Jih-Chao Yeh, Prakash Kishore Hazam, Chu-Yi Hsieh, Po-Hsien Hsu, Wen-Chun Lin, Yun-Ru Chen, Chao-Chin Li, Jyh-Yih Chen

**Affiliations:** a Marine Research Station, Institute of Cellular and Organismic Biology, Academia Sinica, Jiaushi, Ilan, Taiwan; b Institute of Fisheries Science, National Taiwan University, Taipei, Taiwan; c Academia Sinica Protein Clinic, Institute of Biological Chemistry, Academia Sinica, Taipei, Taiwan; d Institute of Cellular and Organismic Biology, Academia Sinica, Nankang, Taipei, Taiwan; e The iEGG and Animal Biotechnology Center and the Rong Hsing Research Center for Translational Medicine, National Chung Hsing University, Taichung, Taiwan; Veterans Affairs Northeast Ohio Healthcare System

**Keywords:** antimicrobial peptide, stapling peptide, polymicrobial sepsis, cecal ligation and puncture, peptide design, Tilapia piscidin 4 (TP4)

## Abstract

In this work, we sought to develop a TP4-based stapled peptide that can be used to counter polymicrobial sepsis. First, we segregated the TP4 sequence into hydrophobic and cationic/hydrophilic zones and substituted the preferred residue, lysine, as the sole cationic amino acid. These modifications minimized the intensity of cationic or hydrophobic characteristics within small segments. Then, we incorporated single or multiple staples into the peptide chain, bracketing the cationic/hydrophilic segments to improve pharmacological suitability. Using this approach, we were able to develop an AMP with low toxicity and notable *in vivo* efficacy.

**IMPORTANCE** In our *in vitro* studies, one dual stapled peptide out of the series of candidates (TP4-3: FIIXKKSXGLFKKKAGAXKKKXIKK) showed significant activity, low toxicity, and high stability (in 50% human serum). When tested in cecal ligation and puncture (CLP) mouse models of polymicrobial sepsis, TP4-3 improved survival (87.5% on day 7). Furthermore, TP4-3 enhanced the activity of meropenem against polymicrobial sepsis (100% survival on day 7) compared to meropenem alone (37.5% survival on day 7). Molecules such as TP4-3 may be well suited for a wide variety of clinical applications.

## INTRODUCTION

Antibiotics are one of the most impactful classes of chemotherapeutic agents discovered to date (1). However, the recent steep rise in multidrug-resistant (MDR) pathogens and the limited number of effective drugs pose major human health concerns (2, 3). Moreover, microbe-induced sepsis remains a life-threatening condition, responsible for an annual mortality of 11 million individuals (4–6). During the COVID-19 pandemic, the majority of deaths were reported to involve virus-mediated sepsis (6). Therefore, the World Health Organization (WHO) has categorized sepsis as a global health priority with insufficient therapeutic options. Current treatments for sepsis only have supportive rather than curative functions (6), so there is a dire need for new effective antibiotics or alternatives. Here, we sought to develop antimicrobial peptides (AMPs) for the treatment of sepsis because these molecules have proven activity against drug-resistant pathogens and/or biofilms (7–10).

In addition to their direct killing of invading pathogens, AMPs may act by modulating immune responses (7, 8). Moreover, AMPs are considered potential alternatives for use against drug-resistant superbugs due to their high selectivity and low degree of induced resistance (9–16). However, their application is limited due to insufficient *in vivo* efficacy (17). Approaches such as end-capping, stapling, unnatural amino acid insertion, and conjugation with lipid moieties (18–27) have been shown to enhance the *in vivo* efficacy of other peptides. Hence, we decided to use sequence optimization and peptide stapling (13, 23, 24) as a design strategy to develop potential AMPs for *in vivo* application.

We selected tilapia piscidin 4 (TP4) as a template based on its broad-spectrum antimicrobial and immunomodulatory properties (28). TP4 is a piscidin AMP isolated from Nile tilapia (Oreochromis niloticus) (28–30). Despite its multipotent activity, TP4 is quite cytotoxic (29, 31), which presents a major obstacle to its clinical application. A key reason for its toxicity is likely the penta-arginine tract near the C terminus of the peptide (32). In addition, the linear peptide is prone to enzymatic degradation in physiological conditions (16, 24). In the current study, we successfully designed stable peptides with significant antimicrobial potency and minimal cytotoxicity through sequence optimization and peptide stapling. In particular, segregated hydrophilic and hydrophobic zones were identified within the peptide sequence, followed by peptide stapling of these regions. We generated several TP4 derivatives and selected TP4-3, an active AMP with low toxicity and improved stability, for *in vivo* studies. In a model of polymicrobial sepsis induced by cecal ligation and puncture (CLP), TP4-3 demonstrated better activity (% survival) than meropenem. Hence, we successfully developed an antimicrobial for *in vivo* application with high efficacy, improved stability, and low off-target toxicity.

## RESULTS

### Design of stapled peptides.

**FIG 1:**
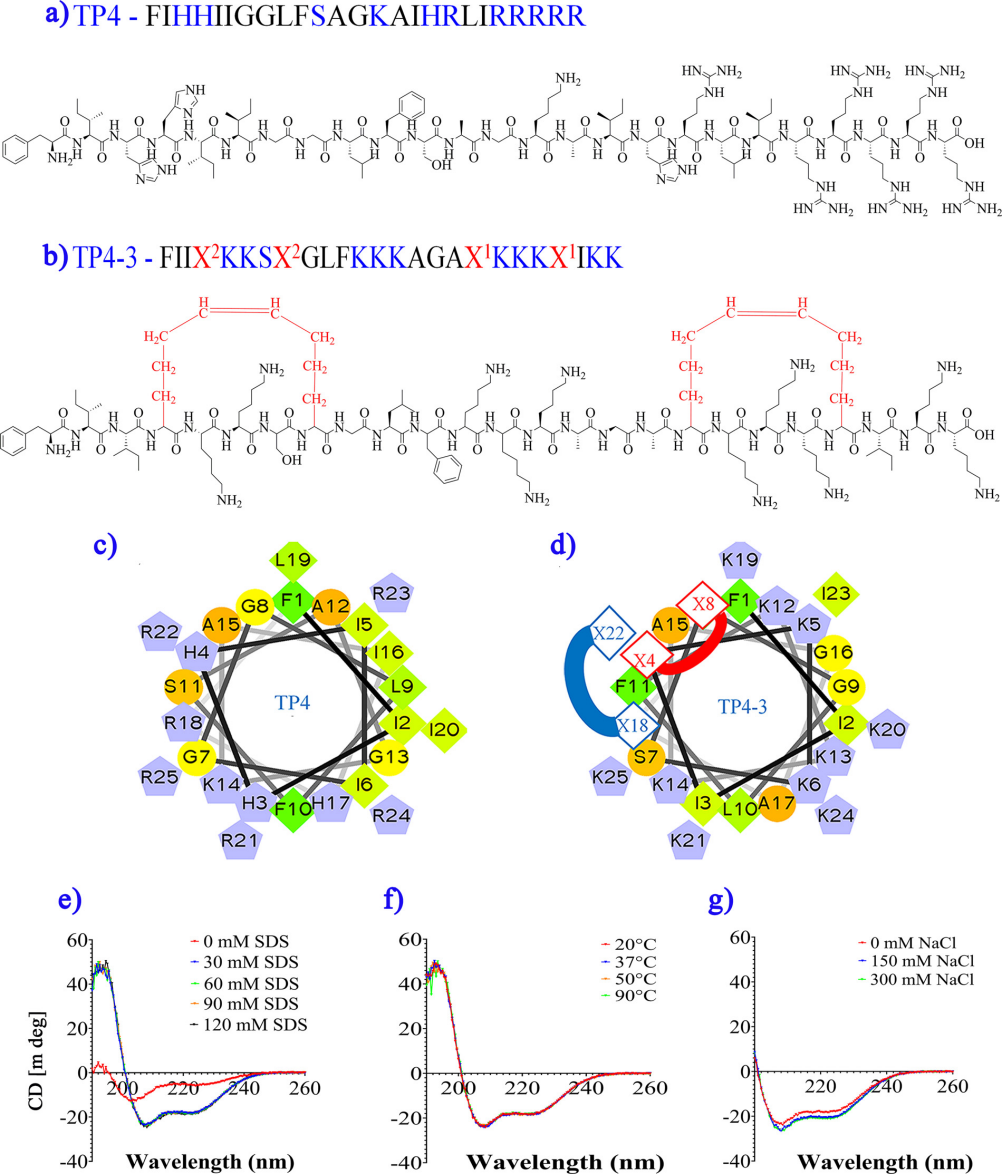
Schematic representation of the (a and b) peptide sequences and structural details of the stapled peptide. Blue letters indicate hydrophilic/cationic amino acids, black letters denote hydrophobic amino acids. A red letter “X” indicates a stapling position, with matching numbers indicating each stapling pair. Stapling pairs are covalently linked through a carbon chain. Helical wheel shows cationic or hydrophobic residues, indicated by single-letter amino acid codes, for (c) TP4 and (d) TP4-3. Stapling sites are shown at X4–X8 and X18–X22. (e) Circular dichroism (CD) spectra of TP4-3 peptide treated with various concentrations of SDS in 10 mM phosphate-buffered saline (PBS) (pH 7.4). At 0 mM SDS (10 mM PBS), the structure appears to be extended, while SDS induces a characteristic pattern for α-helical structure. (f) CD spectra in the presence of 30 mM SDS mixed in 10 mM PBS at different temperatures. (g) Spectral recordings at added salt concentrations ranging from 0 to 300 mM NaCl in the presence of 30 mM SDS in 10 mM PBS.

TP4 is a naturally occurring, highly active AMP, but its application *in vivo* is limited due to its severe toxicity (29–32). To overcome this limitation, we modified the original sequence and tested the derivatives in a hemolysis assay. First, the peptide sequence was segregated into four hydrophobic and three hydrophilic/cationic tracts (Fig. S1 in the supplemental material), which were individually sequestered by stapling across the sequences (Table 1, Fig. 1a and b, Fig. S2). The core idea behind this rearrangement was to minimize the excessive accumulation of either cationic or hydrophobic amino acids within a small segment of peptide, thereby achieving scattered amphipathicity across the sequence. In addition, we substituted all cationic amino acids with lysine because it is highly prevalent in AMPs (33).

**TABLE 1:** TP4-derived stapled peptides, with sequences and molecular masses

Peptide	Sequence and stapling sites^*a*^	S. aureus		A. baumannii		Observed molecular mass (Da)	15% hemolysis concn (μM)^*b*^
		MIC (μM)	MBC (μM)	MIC (μM)	MBC (μM)		
TP4	FIHHIIGGLFSAGKAIHRLIRRRRR	1.56	1.56	3.13	3.13	2,979.8	<3.13
TP4-1	FIIIKKSGGLFKKKAGAX^1^KKKX^1^IKK	>25	>25	>25	>25	2,810.6	>100
TP4-2	FIIIKKSGGLX^2^KKKX^2^GAX^1^KKKX^1^IKK	>25	>25	12.5	12.5	2,842.6	>100
TP4-3	FIIX^2^KKSX^2^GLFKKKAGAX^1^KKKX^1^IKK	>25	>25	3.13	3.13	2,890.7	>100
TP4-4	FIIX^2^KKSX^2^GLX^3^KKKX^3^GAX^1^KKKX^1^IKK	>25	>25	12.5	12.5	2,922.7	>100

a The letter “X”indicates a stapling site, with matching superscript numbers indicating *i* – *i *+* *4 cross-linked pairs.

b Percent hemolysis was measured using human red blood cells. Experiments were repeated twice, with each sample tested in triplicate. The results showed low hemolysis potential of stapled peptide compared with parental TP4.

Next, we introduced single or multiple staples in an *i* to *i + 4* arrangement, enclosing three hydrophilic/cationic amino acids (Fig. 1a and b, Fig. S2). This modification did not alter percent amphipathicity, as the amino acids at the point of stapling (Fig. 1a and b) were all hydrophobic in nature. Thus, we created four stapled peptide sequences: a single-stapled peptide, two double-stapled peptides, and one triple-stapled peptide. Each peptide was synthesized at >95% purity (Fig. S3 to S6).

### Antimicrobial and hemolysis activities of designed peptides.

The antimicrobial activities of the designed peptides were screened against Staphylococcus aureus and Acinetobacter baumannii. TP4-3 had comparable efficacy (3.13 μM) to TP4 when tested against A. baumannii (Table 1). However, the activity of TP4-3 was restricted to Gram-negative species. The MICs of TP4-1 (>25 μM), TP4-2 (12.5 μM), and TP4-4 (12.5 μM) against A. baumannii were all relatively higher than that of TP4-3. All peptides were tested for their hemolytic potential. The lowest tested concentration of TP4 (3.13 μM) showed higher hemolysis than the highest tested concentration (100 μM) of each stapled peptide (Table 1, Fig. S7). Finally, the antimicrobial potencies of the selected peptides (TP4 and TP4-3) were tested in the presence of 50% human serum and 5% lung surfactant (Table 2). TP4-3 retained its activity against A. baumannii, while TP4 was inactive in the presence of 50% human serum. Both the peptides lost their activity in the presence of 5% lung surfactant (Table 2). Because TP4-3 retained its activity in the presence of 50% human serum, we selected this peptide for further evaluation.

**TABLE 2:** MICs and MBCs of active peptides against A. baumannii strains in 50% human serum or 5% lung surfactant^*a*^

Peptide	50% human serum		5% lung surfactant	
	MIC	MBC	MIC	MBC
TP4	>25	>25	>25	>25
TP4-3	3.13	3.13	25	25

a MICs and MBCs are expressed in μM. Experiments were repeated twice, with each sample tested in triplicate.

When tested in Mueller-Hinton (MH) medium, TP4-3 displayed MBC values of 25 μM against Escherichia coli, Pseudomonas aeruginosa, NDM-1 Klebsiella pneumoniae, and Enterobacter aerogenes, and 12.5 μM against Salmonella enterica (Table 3). The control peptide LL-37 showed better activity, and meropenem was significantly active against all species except NDM-1 K. pneumoniae (Table 3). TP4-3 displayed improved activity when tested in the presence of 50% human serum. It has been proposed that physiological factors may limit the potency of AMPs. However, serum itself possesses some antimicrobial potency (34), which may contribute to the net experimental outcome. Importantly, LL-37 lost its activity in serum, whereas meropenem retained its activity against wild-type strains (50%) (Table 3).

**TABLE 3:** MICs and MBCs of TP4-derived peptides toward different bacterial species in MHB medium and 50% human serum containing MHB medium^*a*^

Testing agent	Medium	Inhibitory concn (μM)	Bacterial species				
			E. coli	P. aeruginosa	NDM*-*1 K. pneumoniae	S. enterica	E. aerogenes
TP4	MHB	MIC	3.13	12.5	6.25	6.25	0.78
		MBC	3.13	12.5	6.25	6.25	0.78
	HS	MIC	>25	>25	>25	NA^*b*^	NA
		MBC	>25	>25	>25	NA	NA
TP4-3	MHB	MIC	25	25	25	6.25–12.5	25
		MBC	25	25	25	12.5	25
	HS	MIC	12.5−25	12.5−25	12.5	NA	NA
		MBC	12.5−25	12.5−25	12.5	NA	NA
LL-37	MHB	MIC	6.25	6.25−12.5	25	>25	6.25
		MBC	6.25	6.25−12.5	25	>25	6.25
	HS	MIC	>25	>25	>25	NA	NA
		MBC	>25	>25	>25	NA	NA
Meropenem	MHB	MIC	0.39	3.13	>25	3.13–6.25	0.39
		MBC	0.39	6.25	>25	6.25	0.39
	HS	MIC	0.78	1.56	>25	NA	NA
		MBC	0.78	3.13	>25	NA	NA

a HS, 50% human serum containing MHB medium. Experiments were repeated twice, with each sample tested in triplicate.

b NA (not applicable): S. enterica and E. aerogenes were unable to grow in 50% human serum.

### Antimicrobial activity of TP4-3 tested against MDR *A. baumannii* bacterial species.

The antimicrobial potencies of TP4-3, the control peptides (TP4, LL-37), and meropenem were assessed against a series of 12 MDR A. baumannii strains (Table 4, Table S1). TP4, TP4-3, and LL-37 had significant activities, but meropenem was inactive in the presence of MH medium (Table 4). In addition, the activity of TP4-3 was only marginally reduced, unlike TP4, LL-37, and meropenem, which showed no activity in the presence of 50% human serum (Table 4).

**TABLE 4:** MICs and MBCs of TP4, TP4-3, LL-37, and meropenem against MDR A. baumannii strains in MHB medium and MH medium containing 50% human serum^*a*^

Testing agent	Medium	Inhibitory concn (μM)	MDR A. baumannii strains											
			14B0091	2088	921	1019	1033	1607	1702	2962	2982	2997	2998	3618
TP4	MHB	MIC	<1.56	<1.56	<1.56	<1.56	<1.56	<1.56	<1.56	<1.56	3.13	<1.56	<1.56	3.13
		MBC	<1.56	<1.56	<1.56	<1.56	6.25	<1.56	<1.56	<1.56	3.13	<1.56	<1.56	6.25
	HS	MIC	>25	>25	>25	>25	>25	>25	>25	>25	>25	>25	>25	>25
		MBC	>25	>25	>25	>25	>25	>25	>25	>25	>25	>25	>25	>25
TP4-3	MHB	MIC	3.13	6.25	6.25	12.5	6.25–12.5	6.25	3.13-6.25	6.25	6.25	3.13–6.25	6.25	12.5
		MBC	3.13	6.25	6.25	12.5	6.25–12.5	12.5	3.13–6.25	12.5	6.25	3.13–6.25	12.5	12.5
	HS	MIC	12.5	3.13–6.25	25	25	12.5	12.5	25	12.5	12.5	6.25	6.25	6.25–12.5
		MBC	>25	6.25–12.5	>25	>25	12.5–25	>25	>25	25	>25	12.5	12.5	12.5
LL-37	MHB	MIC	3.13	3.13–6.25	6.25	6.25	6.25	3.13	3.13	6.25	6.25	1.56	6.25	3.13
		MBC	3.13	3.13–6.25	6.25	6.25	6.25	3.13–6.25	3.13	6.25	12.5	1.56	6.25–12.5	3.13
	HS	MIC	>25	>25	>25	>25	>25	>25	>25	>25	>25	>25	>25	>25
		MBC	>25	>25	>25	>25	>25	>25	>25	>25	>25	>25	>25	>25
Meropenem	MHB	MIC	>25	>25	>25	>25	>25	>25	>25	>25	>25	>25	>25	>25
		MBC	>25	>25	>25	>25	>25	>25	>25	>25	>25	>25	>25	>25
	HS	MIC	>25	>25	>25	>25	>25	>25	>25	>25	>25	>25	>25	>25
		MBC	>25	>25	>25	>25	>25	>25	>25	>25	>25	>25	>25	>25

a MDR, multidrug-resistant; HS, MH medium containing 50% human serum. Experiments were repeated twice, with each sample tested in triplicate.

### Robust antimicrobial activity of peptide TP4-3.

The activities of AMPs may be lost under various conditions (35, 36), so we tested the activities of TP4-3 against A. baumannii strains at different pH values, temperatures, and physiological ion concentrations, and under hyperglycemic conditions. TP4-3 retained its activity at pH values ranging from 8 to 10. However, the activity of TP4-3 was reduced 4-fold (MBC) in pH 6 (Table 5). TP4 showed comparable activity within a range of pH 6 to 10, with improved activity at basic pH.

**TABLE 5:** Antimicrobial activity of TP4-3 against A. baumannii under different conditions^*a*^

Testing agent	Inhibitory concn (μM)	pH					Salts and glucose						NM
		4	6	8	10	12	NaCl	KCl	NH_4_Cl	MgCl_2_	CaCl_2_	0.2% glucose	
TP4	MIC	NA	3.13−6.25	1.56−3.13	0.78	NA^*b*^	1.56	3.13	1.56−3.13	3.13	6.25−12.5	1.56−3.13	3.13
	MBC	NA	3.13−6.25	1.56−3.13	0.78	NA	1.56	3.13	1.56−3.13	3.13	6.25−12.5	1.56−3.13	3.13
TP4-3	MIC	NA	12.5	3.13	0.78	NA	6.3	3.13	6.3	12.5	25	3.13	3.13
	MBC	NA	12.5	3.13	3.13	NA	6.3	6.3	6.3	12.5	25	3.13	3.13

a Normal medium (NM [pH 7.4]) was used as a control. Experiments were repeated twice, with each sample tested in triplicate.

b NA (not applicable), bacteria did not grow in media adjusted to pH 4 and pH 12.

TP4 also showed consistent activity in the presence of physiological ions except for CaCl_2_, showing enhanced MBC (6.25 to 12.5 μM), unlike normal media which displayed bactericidal activity at a low concentration of 3.13 μM (Table 5). It is likely that AMPs tend to display compromised activity in the presence of physiological ions (17). In addition, in the case of TP4-3, media supplemented with NaCl, KCl, or NH_4_Cl lowered the bactericidal potency of TP4-3 (MBC) by up to 2-fold compared to non-supplemented medium (Table 5). Similarly, media supplemented with MgCl_2_ and CaCl_2_ caused up to 4-fold and 8-fold lower bactericidal potency of TP4-3, respectively. Glucose (0.2%) did not affect the activity of TP4-3 (Table 5). In addition, TP4-3 retained its activity, even when pre-incubated at temperatures ranging from 40 to 80°C, but it had reduced activity after incubation at 100°C. TP4 retained similar activity across all incubation temperatures. Meropenem showed a similar profile but completely lost activity after incubation at 100°C (Table 6). Furthermore, TP4-3 retained its activity for up to 6 h of serum pretreatment. Afterward, the activity was reduced by 2-fold (12 h) or 4-fold (24 h). In contrast, TP4 lost its activity in the presence of 50% human serum. Of note, meropenem retained its activity for up to 12 h of serum pretreatment. The activity was lowered by 2-fold after 24 h pre-incubation in 50% human serum (Table 6).

**TABLE 6:** Antimicrobial activity of TP4-3 and meropenem against A. baumannii after pre-exposure to various temperatures (40, 60, 80, and 100°C) for 1 h^*a*^

Testing agent	Inhibitory concn. (μM)	Temp (°C)				Preincubation time in 50% human serum (h)			
		40	60	80	100	2	6	12	24
TP4	MIC	1.56	1.56	1.56	1.56	>25	>25	>25	>25
	MBC	1.56	1.56	1.56	1.56	>25	>25	>25	>25
TP4-3	MIC	6.25	6.25	6.25–12.5	12.5–25	3.13	3.13	6.25	>25
	MBC	6.25	6.25	6.25–12.5	12.5–25	3.13	3.13	6.25	6.25−12.5
Meropenem	MIC	6.25	6.25	12.5	>25	6.25	6.25	6.25	12.5
	MBC	6.25	6.25	12.5	>25	6.25−12.5	6.25−12.5	12.5	25

a Antimicrobial activity of each agent was also measured after pre-exposure to 50% human serum for various durations. Experiments were repeated twice, with each sample tested in triplicate.

Based on our results up to this point, we concluded that TP4-3 shows significant activity and low hemolysis. The peptide also retains its activity in the presence of physiological ions, with human serum, at various pH values, and over a broad range of temperatures. Therefore, TP4-3 was selected for further analyses.

### Structural analysis of stapled peptides.

The peptide TP4-3 contains small segments of hydrophobic and cationic amino acids, which are distributed across the helical wheel (Fig. 1c and d, Fig. S8). Notably, TP4-3 exhibited an α-helical structure in the presence of sodium dodecyl sulfate (SDS), but it had an extended structure in buffer alone (Fig. 1e). The α-helical structure was indicated by negative peaks at roughly 208 and 222 nm and a positive peak at 190 nm. Incubations at different temperatures (Fig. 1f) and salt concentrations (Fig. 1g) had no effect on the helical structure in the presence of 30 mM SDS-containing buffer. Changes to the amino acid composition were made based on previous studies (33), and these changes proved to be influential, as one modified peptide (TP4-3) showed notable activity in the presence of 50% human serum, unlike TP4 (Table 2). Moreover, the influence of our structural rearrangements, preferred amino acid content, and stapling on the activity of TP4-3 in the presence of human serum suggested that the peptide had probable *in vivo* activity. It is important to note that the location of stapling was crucial, as peptides with similar compositions and numbers of stapling sites (TP4-2, TP4-3) showed different efficacies (Table 1, Fig. 1, Fig. S2). Hence, the overall construction of the peptide was critical to its function because the final structure is the net outcome of the overall composition.

### Bacteria killing kinetics and serial passage assay.

**FIG 2:**
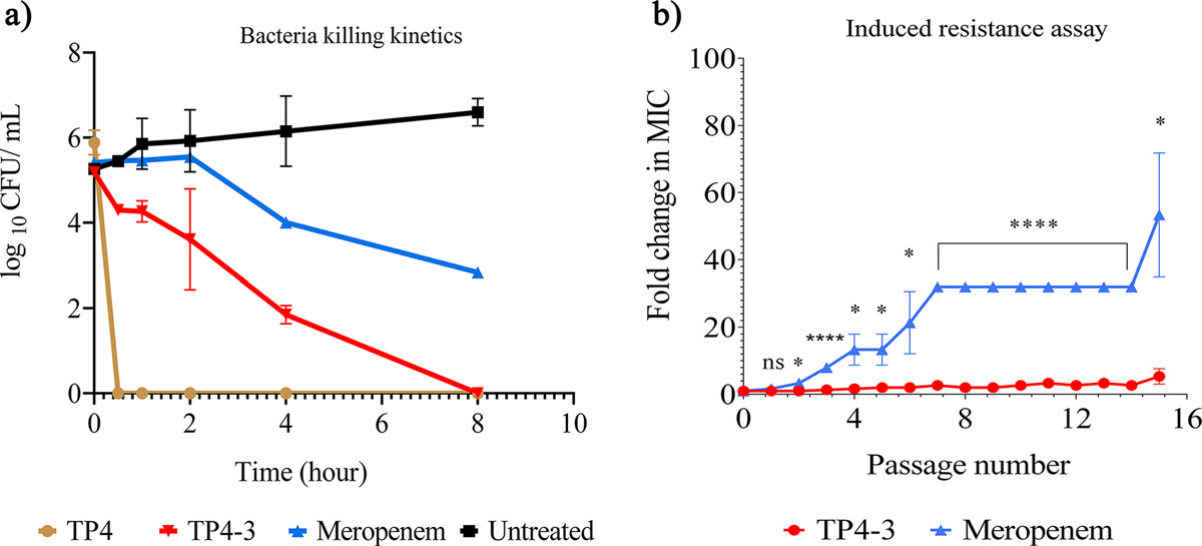
(a) Bactericidal potencies of the peptides TP4 and TP4-3 and meropenem against Acinetobacter baumannii as a function of time at their respective MBCs. (b) Estimation of the fold change in the MICs of TP4-3 and meropenem based on induced resistance assay. Experiments were repeated three times, with each sample tested in triplicate. *, *P* < 0.05; ****, *P* < 0.0001; ns, no statistical significance.

Next, we performed bacteria-killing kinetics assays. In these experiments, TP4-3 cleared the entire bacterial load within 8 h, whereas meropenem could reduce approximately half of the bacterial population within the same time (Fig. 2a). TP4 cleared the entire bacterial load within 30 min. Overall, the bactericidal potency of TP4-3 was similar to that of meropenem, but with half of the exposure time. In addition, the serial passage assay revealed that a very low degree of induced resistance resulted from exposure to TP4-3, unlike with meropenem. TP4-3 displayed a net increase in the MIC of 5-fold after serial passage for 15 cycles, whereas the MIC of meropenem was increased by 53-fold after a similar number of cycles (Fig. 2b). Hence, prolonged TP4-3 exposure stimulated less induced resistance than meropenem.

### Antibiofilm potential of TP4-3 against *A. baumannii*.

**FIG 3:**
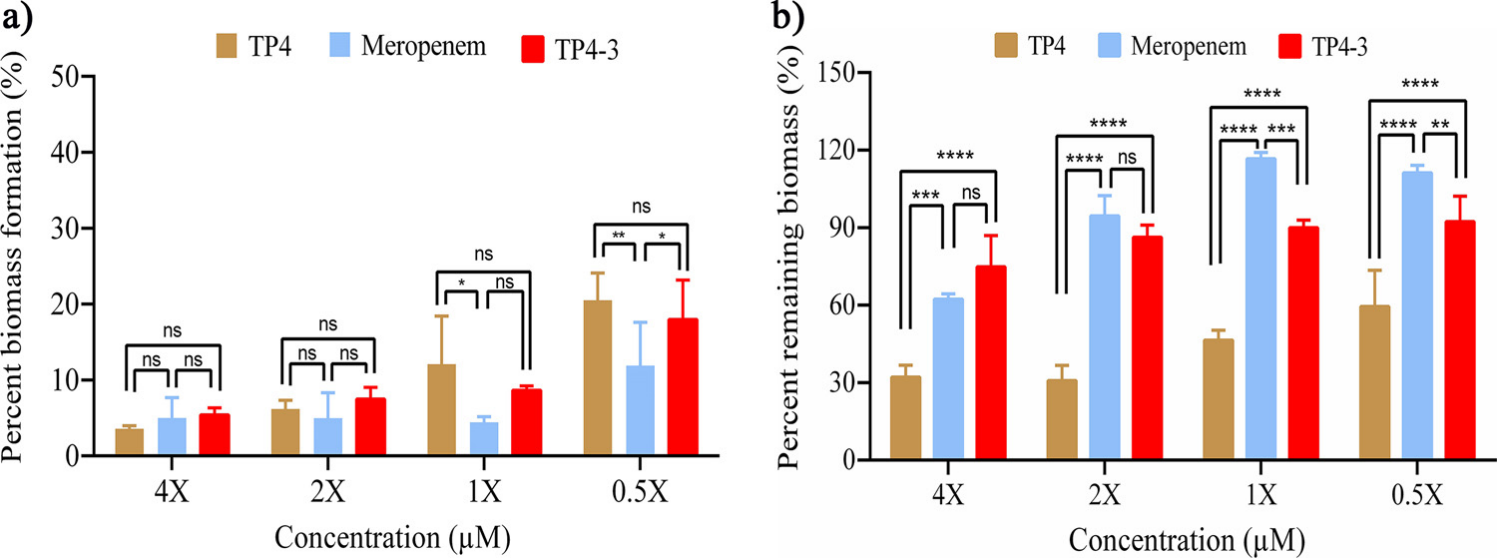
Antibiofilm activities of TP4, TP4-3, and meropenem against A. baumannii biofilm ranging from 0.5× to 4× MIC of test candidates. (a) Inhibition of biofilm growth by TP4, TP4-3, and meropenem at various concentrations. (b) Mature biofilm rupture potential of TP4, TP4-3, and meropenem. Experiments were repeated three times, with each sample tested in triplicate.

We tested the antibiofilm potential of TP4, TP4-3, and meropenem against A. baumannii biofilms over concentrations ranging from 0.5× to 4× MIC of the test candidates. TP4, TP4-3, and meropenem all showed substantial inhibition of biomass formation (Fig. 3a). However, TP4, TP4-3, and meropenem showed minor potential for causing biofilm rupture even at 4× MIC. TP4 showed the best rupture potency among the treatments (Fig. 3b), while TP4-3 displayed better biofilm-rupturing potential than meropenem (Fig. 3b).

### Fractional inhibitory concentration index of TP4-3 and meropenem/doxycycline.

We next tested the activity of TP4-3 in combination with meropenem or doxycycline (Table S2, see supplemental material). The results showed that TP4-3 and meropenem mostly had additive effects (fractional inhibitory concentration index [FICI] < 1) when used against MDR A. baumannii or NDM-1 K. pneumoniae strains. However, combined treatments of TP4-3 and meropenem acted synergistically against the MDR strains A. baumannii 921, A. baumannii 1019, and A. baumannii 3618. Similarly, combinations of TP4-3 and doxycycline showed additive effects (FICI < 1) against most of the tested MDR A. baumannii strains. However, the combination showed synergistic potential against MDR A. baumannii 1019 and NDM-1 K. pneumoniae strains. Importantly, both combinations showed synergistic activities against wild-type bacterial strains (Table S2).

### Mechanism of TP4-3 bactericidal activity.

**FIG 4:**
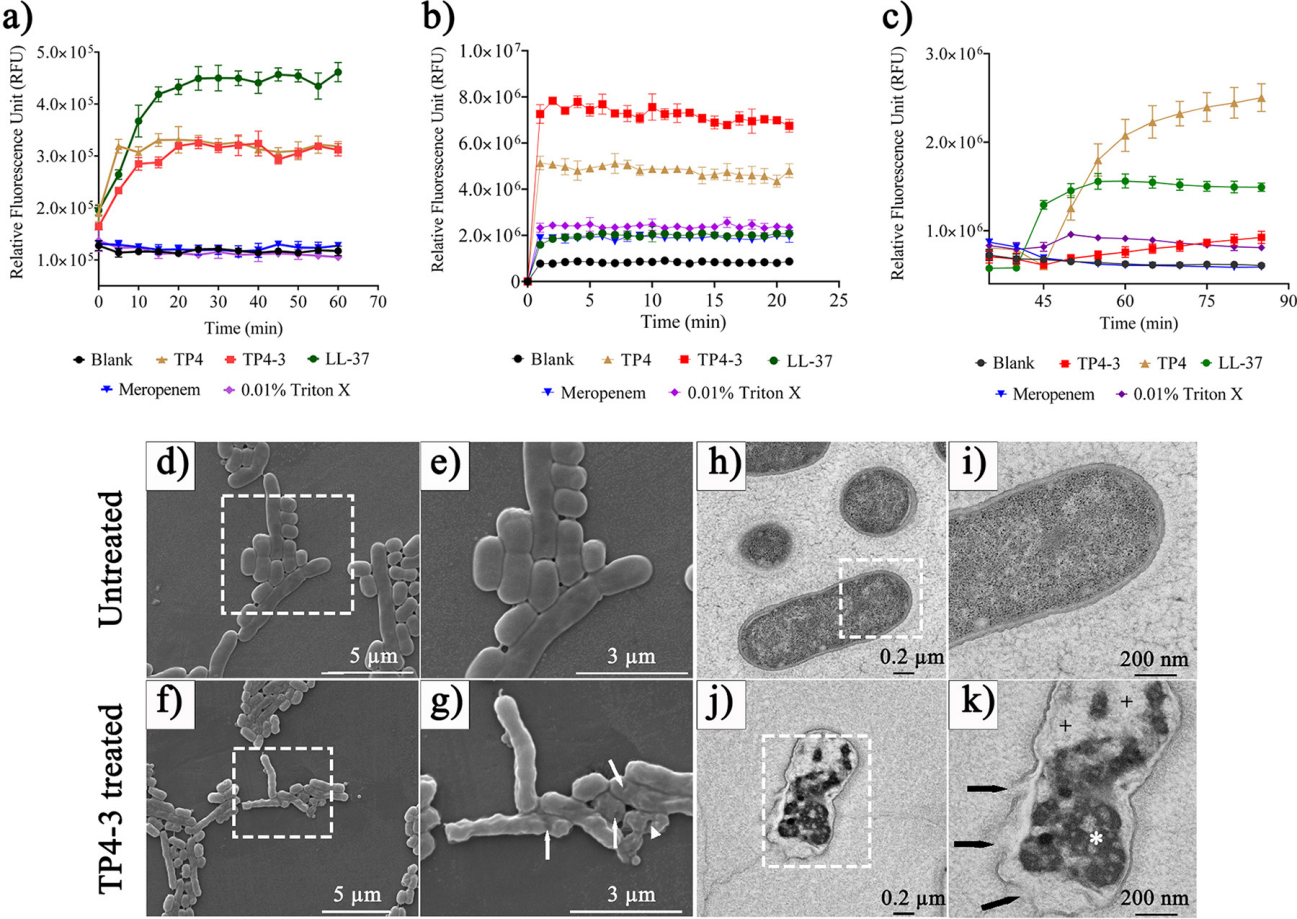
Membranolytic activity of TP4-3 as tested with (a) propidium iodide (PI) and (b) *N*-phenyl 1-naphthylamine (NPN). Higher fluorescence signal indicates higher membrane lysis and increased dye uptake within the bacterial cells. (c) Bacterial membrane depolarization activity of TP4-3 using DiBAC_4_(3) [bis-(1,3-dibarbituric acid)-trimethine oxanol] dye. TP4, LL-37, and meropenem served as controls. Experiments were repeated three times, with each sample tested in triplicate. (d to k) Qualitative assessment of TP4-3 membranolytic potential against A. baumannii strains using scanning electron microscopy (SEM; f and g) and transmission electron microscopy (TEM; j and k). TP4-3-treated samples at 1× MIC had deformed bacterial membrane architecture compared with untreated membranes in SEM (d and e) and TEM (h and i) images, which showed no deformation. Selected areas in white squares are enlarged for better comparison of SEM and TEM images. White arrows indicate membrane deformation, whereas white triangle shows the formation of prominent holes in SEM images (c and d). Ruptured or dissolved membrane architecture is shown by the black arrow in TEM images (g and h); white asterisks indicate aggregated intracellular components. Black plus sign (+) shows hollow areas formed due to peptide (TP4-3) treatment. Scale bars in SEM images = 3 μm and 5 μm. In TEM images, scale bars = 0.2 μm and 200 nm.

We tested the mechanism of bactericidal activity using propidium iodide (PI) and *N*-phenyl 1-naphthylamine (NPN) fluorescent dyes. A. baumannii bacterial cells (10^8^ CFU/mL) were treated with 25 μM TP4-3, 25 μM meropenem, and Triton X-100 at 0.01%. The bacterial cells treated with TP4-3 showed fluorescence signals that were approximately 3- and 4-fold higher than the controls for PI and NPN, respectively, indicating maximum membrane lysis (Fig. 4a and b).

In addition, TP4-3 treatment caused a substantial rise in the fluorescence signal from DiBAC4(3) [bis-(1,3-dibarbituric acid)-trimethine oxanol] compared to the blank control, indicating the induction of membrane depolarization. The signals from meropenem- and Triton X-100-treated bacteria were reduced after prolonged exposure (Fig. 4c). TP4 caused similar PI fluorescence as TP4-3, but its NPN signal was lower, and the DiBAC4(3) signal was higher than TP4-3-treated cells. In addition, we performed scanning electron microscopy (SEM) and transmission emission microscopy (TEM) to provide qualitative validation of the membranolytic potential of the test peptide/drugs. Based on these analyses, TP4-3-treated bacteria (1× MIC) showed significant membrane deformation in contrast to the intact architecture of untreated bacterial membranes (Fig. 4d to k).

### *In vitro* and *in vivo* toxicity of TP4-3.

**FIG 5:**
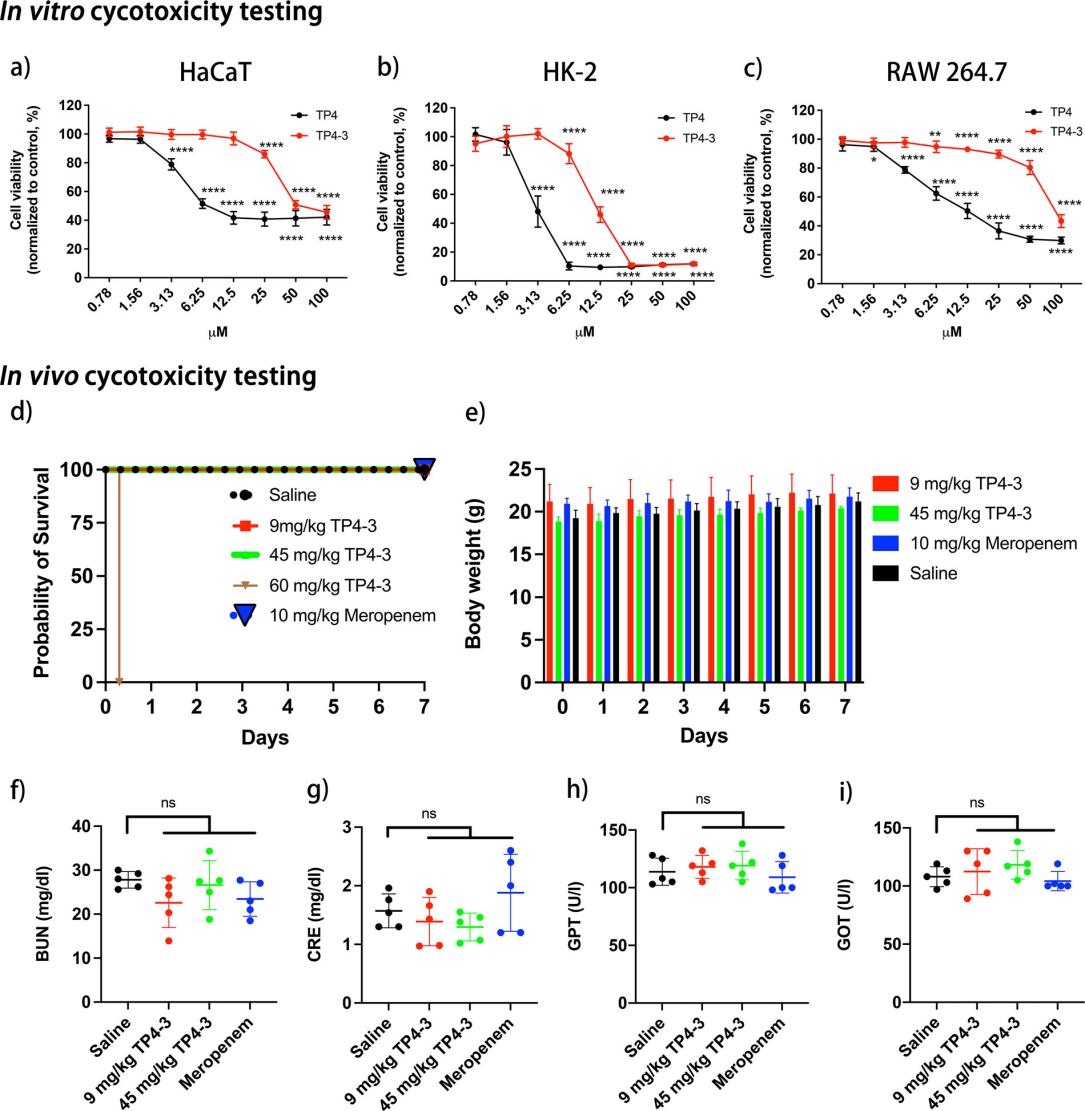
*In vitro* and *in vivo* toxicity of peptide TP4-3. The cytotoxicity of TP4-3 was determined using (a) human keratinocytes (HaCaT), (b) human kidney cells (HK2), and (c) mouse macrophages (RAW 264.7) according to an alamarBlue assay. Cell viability was normalized to the control group. All measurements were performed in triplicate. *In vivo* toxicity was assessed using female C57BL/6 mice (*n* = 5) after intravenous administration of TP4-3 (9, 45, and 60 mg/kg), meropenem (10 mg/kg), or saline. (d) Probability of survival, (e) body weight, (f) blood urea nitrogen (BUN) concentration, (g) creatinine (CRE) level, (h) glutamic-pyruvic transaminase (GPT) activity, and (i) glutamic oxaloacetic transaminase (GOT) activity were measured to evaluate the *in vivo* toxicity of the test peptide. Error bars represent mean ± standard deviation (SD). *, *P* < 0.05; **, *P* < 0.01; ***, *P* < 0.001; ****, *P* < 0.0001, ns, no statistical significance.

The cytotoxic potentials of TP4-3 and TP4 were tested using human keratinocyte (HaCaT), human kidney cell (HK-2), and murine macrophage (RAW 264.7) cell lines (Fig. 5a to c). Based on alamarBlue assays, TP4-3 showed <10% cell death at 3.13 μM for HaCaT cells, 3.13 μM for HK-2 cells, and 12.5 μM for RAW 264.7 macrophage cells. However, TP4 caused substantial cell death for all cell lines at very low concentrations (0.78 μM in HaCaT cells, 1.56 μM in both HK2 and RAW 264.7 macrophage cells). We further investigated the *in vivo* toxicity of TP4-3 in female mice (C57BL/6) at intravenous (i.v.) doses of 9 or 45 mg/kg. All treated animals survived for 7 days (Fig. 5d). No significant changes in body weight were observed for any group (Fig. 5e). Furthermore, TP4-3, meropenem, and saline treatments produced no obvious effects on kidney (blood urea nitrogen [BUN] and creatine [CRE]) and liver (serum glutamic oxaloacetic transaminase [GOT] and serum glutamic pyruvic transaminase (GPT)) function markers, suggesting that TP4-3 at i.v. doses up to 45 mg/kg causes no detectable renal or hepatic damage (Fig. 5f to i). However, mice treated with 60 mg/kg TP4-3 died within 10 min of treatment.

### *In vivo* efficacy of TP4-3 in CLP-induced polymicrobial sepsis model.

**FIG 6:**
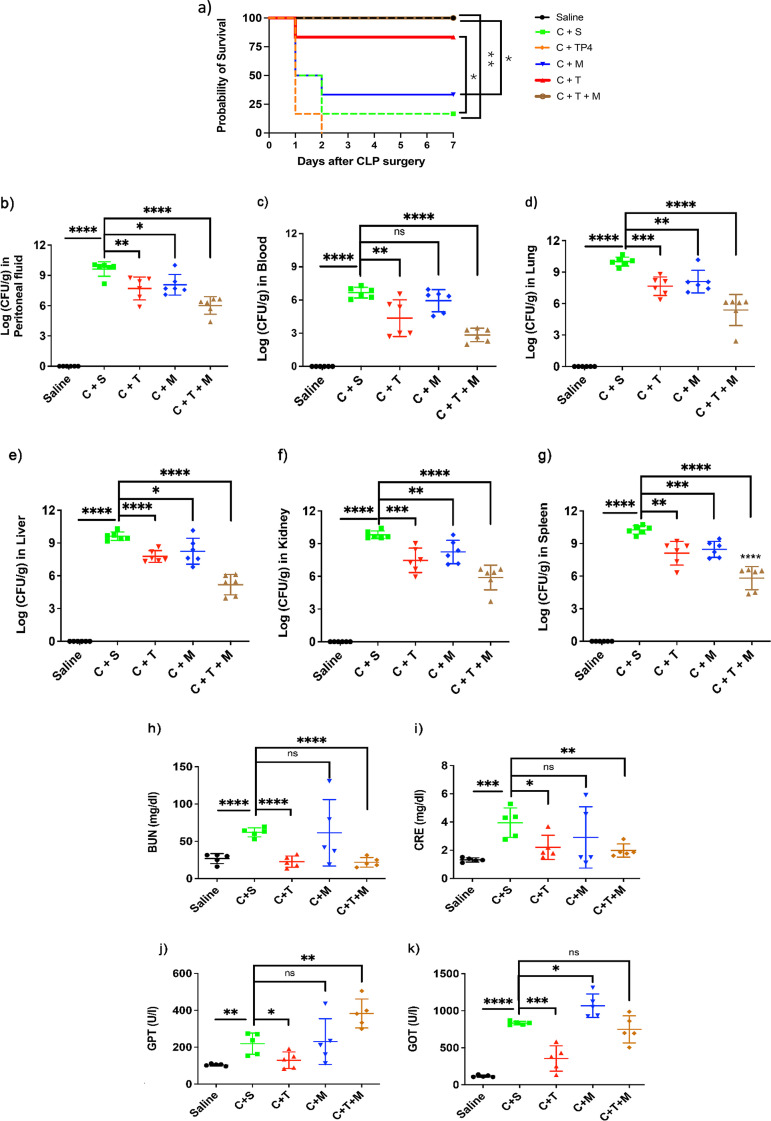
Protective effects of TP4-3 on lethality, bacterial burden, kidney and liver function, and cytokine levels after cecal ligation and puncture (CLP). (a) CLP surgery was performed on female C57BL/6 mice. Thirty minutes after surgery, mice received intraperitoneal injections of saline (C + S), TP4 (4.5 mg/kg, C + TP4), TP4-3 (9 mg/kg, C + T), meropenem (10 mg/kg, C + M), or TP4-3 and meropenem (9 mg/kg TP4-3 and 5 mg/kg meropenem, C + T + M) (*n* = 8). Survival was monitored daily for 8 days. Mice treated with test and standard drugs were monitored, and 24 h after the surgery, (b) peritoneal fluid, (c) blood, (d) lungs, (e) livers, (f) kidneys, and (g) spleens were collected and plated for analysis (*n* = 6). (h) BUN concentration, (i) CRE level, (j) GPT activity, and (k) GOT activity were also measured from serum samples to evaluate kidney and liver function (*n* = 5). *, *P* < 0.05; **, *P* < 0.01; ***, *P* < 0.001; ****, *P* < 0.0001; ns, no statistical significance.

C57BL/6 female mice were subjected to CLP surgery to induce polymicrobial sepsis. Thirty minutes after the surgery, mice received intraperitoneal treatment with TP4-3, meropenem, TP4, or saline. Importantly, the CLP + TP4-3-treated group had markedly less sepsis-induced mortality at 7 days post-operation compared to the CLP + saline group (87.5% versus 12.5% survival, *P* = 0.034, Fig. 6a).

There was no significant difference in mortality between the CLP + meropenem and CLP + saline groups (37.5% versus 12.5% survival, *P* = 0.657). Interestingly, at day 7, the combination treatment of TP4-3 and meropenem after CLP had the highest efficacy compared to CLP + saline (100% versus 12.5% survival, *P* = 0.0047), and it greatly outperformed the CLP + meropenem treatment (100% versus 37.5% survival, *P* = 0.0178). In terms of bacterial survival and growth 24 h after treatment, TP4-3 markedly attenuated the CLP-induced bacterial burdens in the blood, peritoneal lavage fluid, liver, lung, kidney, and spleen compared to the CLP + saline group (Fig. 6b to g). In addition, at 72 h post-treatment, TP4-3 led to lower bacterial burdens in the blood, lung, and liver compared to those in the CLP + saline group (Fig. S9). Interestingly, the bacterial burden in peritoneal lavage fluid at 72 h after TP4-3 treatment showed no significant difference from the measurements at 24 h (*P* = 0.66). However, compared to the 24-h measurements, bacterial burdens were significantly decreased in other evaluated tissues 72 h after TP4-3 treatment (blood: 54% decrease, *P* = 0.048; kidney: 51% decrease, *P* = 0.0025; lung: 51% decrease, *P* = 0.0013; liver: 61% decrease, *P* < 0.0001; spleen: 55% decrease, *P* = 0.0009). Moreover, the group receiving combined treatment with TP4-3 and meropenem (Fig. S10a to f) had even lower bacterial burdens in evaluated tissues compared to CLP mice treated with either TP4-3 or meropenem alone at 24 h post-treatment (Fig. 6b to g).

In addition, we tested the combined efficacy of TP4-3 and meropenem at 72 h after the treatment, finding significantly lower bacterial burdens in the blood, lung, and liver compared to the CLP + saline group (Fig. S9). The bacterial burdens in peritoneal fluid, kidney, and spleen showed no significant differences compared to the CLP + saline treatment. Moreover, the bacterial burdens in all evaluated organs at 72 h after TP4-3 + meropenem treatment showed no significant differences from the measurements at 24 h.

The CLP surgery caused serious kidney and liver dysfunction at 24 h post-operation (Fig. 6h to k). However, TP4-3 significantly improved kidney (BUN and CRE) and liver function (serum glutamic oxaloacetic transaminase [GOT] and serum glutamic-pyruvic transaminase [GPT]) at the 24-h time point (Fig. 6h to k). In contrast, both meropenem- and saline-treated groups still had poor liver function profiles. Thus, TP4-3 does not exacerbate renal and hepatic damage induced by CLP surgery. Furthermore, the combination treatment of TP4-3 and meropenem may improve kidney function (Fig. S10g and h), with compromised liver function (Fig. S10i and j).

**FIG 7:**
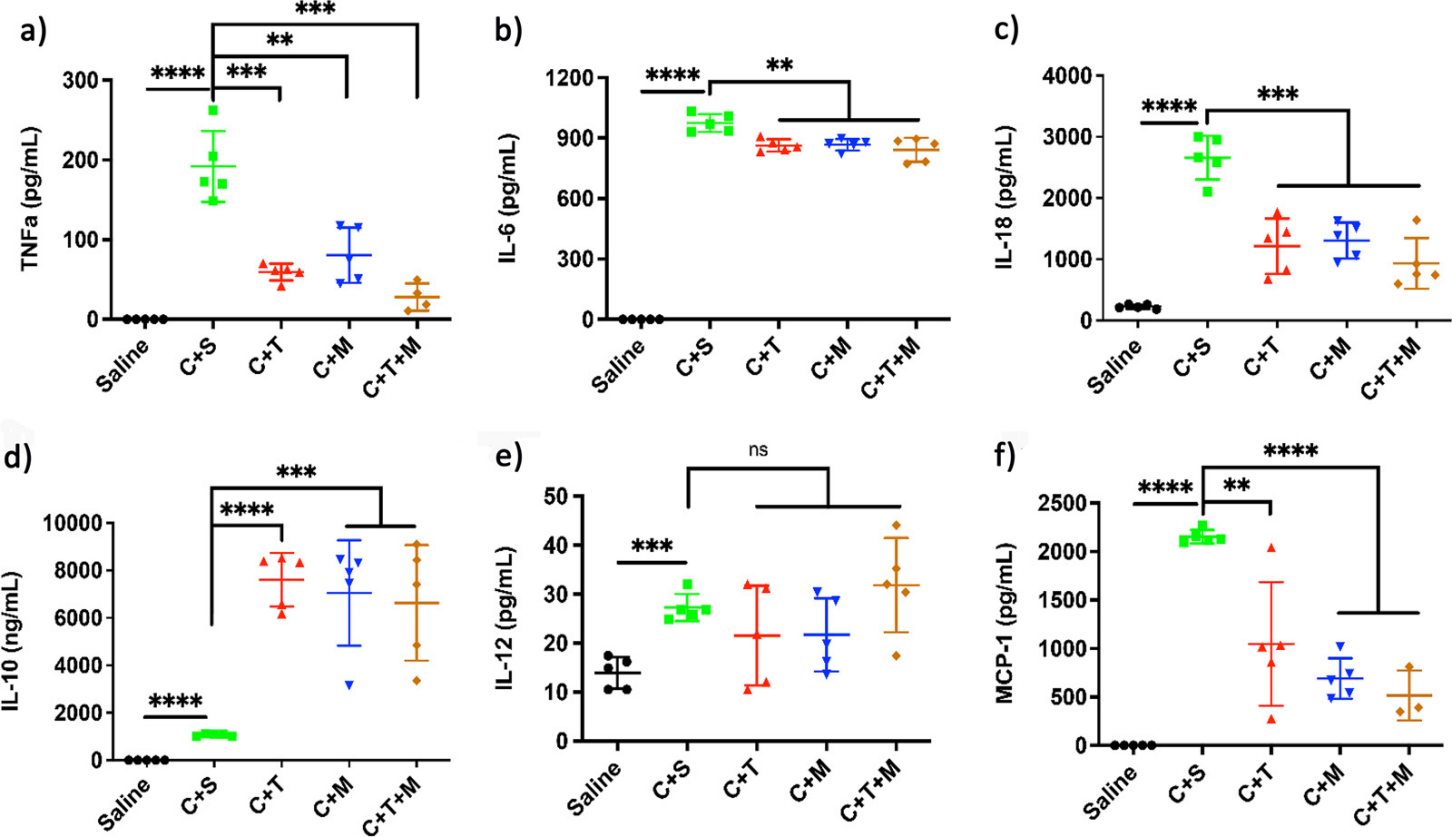
Cytokine levels were quantified for (a) tumor necrosis factor α (TNF-α), (b) interleukin (IL)-6, (c) IL-18, (d) IL-10, (e) IL-12, and (f) monocyte chemoattractant protein 1 (MCP-1); cytokines were measured from serum collected at 24 h using an enzyme-linked immunosorbent assay (ELISA) (*n* = 5). *, *P* < 0.05; **, *P* < 0.01; ***, *P* < 0.001; ****, *P* < 0.0001; ns, no statistical significance.

Hence, our assays up to this point suggested that TP4-3 has the best safety profile among the tested treatments. Because AMPs are known to play important immunomodulatory roles by altering cytokine levels, we evaluated the levels of pro- and anti-inflammatory cytokines (Fig. 7). Compared to the saline-treated group, TP4-3-treated animals had lower levels of tumor necrosis factor α (TNF-α), interleukin (IL)-6, IL-18, and monocyte chemoattractant protein 1 (MCP-1), and a higher level of IL-10; no difference in IL-12 levels was observed (Fig. 7a to f). The CLP mice receiving combined TP4-3 and meropenem treatment (Fig. S10k to p) exhibited similar profiles to the mice receiving TP4-3 treatment alone. These results suggest that TP4-3 alone or in combination with meropenem can effectively prevent the development of polymicrobial sepsis and modulate immune response in mice.

## DISCUSSION

In the current study, we developed an AMP for potential use against sepsis. We began by utilizing TP4, a broad-spectrum but hemolytic AMP (30) (Table 2), as a template molecule. Based on the structure of TP4, we hypothesized that segregating its hydrophobic and hydrophilic/cationic segments might minimize its toxicity (22, 33). In addition, we utilized lysine as the only cationic amino acid due to its high prevalence in natural AMPs (33). Finally, we included stapling (*i* – *i *+* 4*) to enclose the cationic segments in the sequences (Table 1, Fig. 1, Fig. S2) to enhance stability of the peptides.

One test peptide (TP4-3) showed significant bactericidal potency (Table 1), low hemolysis (Table 1, Fig. S7), and extended stability (Table 2). We observed an important correlation between stapling location and numbers, which dictated the overall antimicrobial potency. Despite similar stapling numbers between TP4-2 and TP4-3, the latter showed superior activity. We assume that the given amino acid composition and stapling location was optimal for TP4-3. In relation to amino acid composition, the second stapling in TP4-2 was performed by replacing phenylalanine (F) and alanine (A); whereas the second stapling in the case of TP4-3 was achieved by substituting isoleucine (I) and glycine (G). Hence, we observed a minimal yet distinct variation (Table 1). We assume that the optimization of TP4-3 composition is sequence-specific and may vary with further changes. The other sequences with lower (TP4-1) or higher stapling numbers (TP4-4) displayed lower activity. This may be due to variations in amino acid composition at their stapling site which determine their overall activity.

Thus, our peptide modifications led to minimal hemolysis and enhanced stability, as stapling improves a peptide’s ability to withstand enzymatic degradation (22, 23). TP4-3 displayed a low degree of activity against a series of wild-type Gram-negative bacteria and NDM-1 K. pneumoniae (Table 3). Interestingly, TP4-3 showed better activity against the same series of bacteria in the presence of 50% human serum (Table 3). The test peptide also displayed notable potency against a series of MDR A. baumannii strains, even in the presence of 50% human serum (Table 4). In previous studies, similar outcomes may have been observed due to the presence of serum factors (9, 21, 34). The control peptide (LL-37) lost its activity in the presence of 50% human serum, whereas meropenem was ineffective against MDR strains but had notable activity against wild-type strains (Tables 3 and 4). TP4 displayed notable activity in medium, but its activity was negligible in the presence of 50% human serum. Since TP4-3 retained its activity in the presence of 50% human serum (Table 4), we suspected that the peptide would display antimicrobial activity in *in vivo* models. TP4-3 showed rapid bacterial clearance and a lower degree of induced resistance compared to meropenem (Fig. 2). Notably, TP4-3 displayed significant biofilm inhibition along with meropenem and TP4, showing better biofilm-rupturing potential than meropenem at lower concentrations, but lower activity than TP4 (Fig. 3). Because TP4 application is limited by its toxicity and instability, the anti-biofilm property of TP4-3 could be an important application. It is also noteworthy that TP4-3 acts by causing bacterial membrane lysis (Fig. 4), similar to other AMPs (26). TP4-3 was also active in basic pH, at elevated temperature, and when pre-incubated in serum (for various durations), but it had low activity in the presence of cations (Table 6). TP4-3 also showed additive or synergistic properties when combined with meropenem or doxycycline (Table S2). Perhaps this synergy is not surprising, as AMPs are known to display combinatorial effects with conventional antibiotics (10, 11). Based on our *in vitro* studies, we found that TP4-3 possesses promising activity, enhanced stability, and low hemolytic potential compared to TP4. Therefore, we further tested the compound in a mouse model of CLP-induced sepsis. Previously evaluated AMPs, such as Epi and Pep19-2.5, can effectively neutralize pathogen-associated outcomes, which makes them preferred antimicrobial alternatives (37–39). TP4-3 displayed low toxicity in mice (Fig. 5a to i) at doses up to 45 mg/kg (Fig. 5d). However, we used a low dose of TP4-3 (9 mg/kg) when testing its *in vivo* activity against CLP-induced polymicrobial sepsis. Importantly, TP4-3 prevented the development of sepsis, as measured by mortality, renal and liver dysfunction, and bacterial burden (Fig. 6a to g). In addition, TP4-3 reduced the levels of serum pro-inflammatory cytokines (TNF-α, IL-6, and IL-18) and increased the level of anti-inflammatory IL-10 after CLP surgery. Together, these results suggest that the broad-spectrum antimicrobial and immunomodulatory activities of peptide TP4-3 may be protective against sepsis (Fig. 7). Furthermore, combined treatment with TP4-3 and meropenem prevented mortality and reduced bacterial burdens compared to meropenem treatment alone (Fig. 6, Fig. S9 and S10). Thus, we expect that TP4-3 may be useful to rapidly reduce infection-related complications, retain its potency for a longer duration, and enhance the effectiveness of meropenem treatment for sepsis.

Overall, we conclude that our current strategy of redesigning natural peptides can lower their toxicity while maintaining their *in vivo* activity. Hence, this approach could be utilized to develop AMPs for *in vivo* application.

## MATERIALS AND METHODS

### Peptide synthesis, purification, and characterization.

The designed peptides were synthesized using fluorenylmethoxycarbonyl (F-moc) solid-phase peptide synthesis (GL Biochem Ltd., Shanghai, China) using a previously reported method (23). Peptide stapling was performed by placing Fmoc-(R)-2-(4-pentenyl) alanine at *i* to *i + 4* positions for *i* – *i *+* *4 stapling. A solution of Grubbs first-generation ruthenium catalyst in dichloroethane was added to the peptide resin conjugate, and this step was repeated until maximum stapling was achieved (23). Synthesized peptides were cleaved from the resin by addition of trifluoroacetic acid, followed by precipitation using a 1:1 mixture of ether and hexane. The synthesized peptides were then purified using reverse-phase liquid chromatography over a 25-min gradient from 25% acetonitrile acid in water to 50% acetonitrile with 0.1% trifluoroacetic acid; absorbance detection was performed at 220 nm. The collected peptide fractions were characterized by electrospray ionization mass spectrometry.

### Circular dichroism spectroscopy.

Secondary structure analysis was performed using circular dichroism (CD) spectroscopy. The peptides were dissolved in 10 mM phosphate buffer (pH 7.4) followed by spectroscopic analysis in different SDS concentrations. Peptides were dissolved in the lowest concentration of SDS in phosphate-buffered saline (PBS) to induce α-helical structure formation, and spectra were recorded at various temperatures and salt concentrations (40).

### Antimicrobial assay, serum and lung surfactant sensitivity assay.

The antimicrobial assay was performed according to a modified National Committee for Clinical Laboratory Standards (NCCLS) protocol (41, 42). Briefly, 5 × 10^5^ CFU/mL bacterial cells were treated with various peptide concentrations and incubated at 37 ± 2°C for 16 to 20 h. The lowest concentration with clear wells was recorded as the MIC for each test molecule. The clear wells were further plated in Mueller-Hinton agar (MHA) plates, followed by incubation overnight at 37 ± 2°C. After plating, the lowest concentration at which no growth was observed was recorded as the MBC. For the serum sensitivity assay, the antimicrobial activity of the peptides was tested in medium containing 50% human serum (43, 44).

### Antimicrobial assay in the presence of ions, glucose, and varying pH and temperature.

The antimicrobial assay was performed in the presence of physiological ions and glucose. The test media were supplemented with MgCl_2_ (1 mM), CaCl_2_ (2 mM), NaCl (150 mM), KCl (4.5 mM), NH_4_Cl (6 μM) (45, 46), or glucose (0.2%) (47), followed by routine antimicrobial assay as described previously. For the pH sensitivity assay, the antimicrobial assay was performed in media adjusted to pH values ranging from 4 to 12. To estimate the effects of temperature on antimicrobial activity, the peptide was incubated for 1 h at temperatures ranging from 40 to 100°C (48), followed by the antimicrobial assay as described above.

### Hemolytic assay.

The hemolytic assay was performed after approval from the institutional ethics committee (Academia Sinica Institutional Review Board [IRB]; ID no. AS-IRB02-111075). Blood was collected from healthy humans, and 50 μL of a 2% blood cell suspension in normal saline was treated with an equal volume of peptide at different concentrations. The mixture was then incubated at 37 ± 2°C for 1 h. Next, the cell suspension was centrifuged at 1,000 × *g* for 10 min. Percent hemolysis was estimated by the absorbance of treated and control supernatant samples at 540 nm (23, 44).

### Cytotoxicity assay.

**(i) Cell culture.** Human skin keratinocytes (HaCaT), human kidney (HK-2), and RAW 264.7 macrophage cells were obtained from the Bioresource Collection and Research Center (BCRC, Hsinchu, Taiwan). HaCaT and RAW 264.3 macrophage cells were cultured in Dulbecco’s modified Eagle’s medium (DMEM, Thermo Fischer, MA, USA.) supplemented with 10% FBS, penicillin (100 U/mL), and streptomycin (100 μg/mL) at 37°C under 5% CO_2_. HK-2 cells were cultured in glucose-free Keratinocyte serum-free medium (K-SFM) supplemented with 0.05 mg/mL bovine pituitary extract (BPE), 5 ng/mL EGF, 100 U/mL penicillin, and 100 mg/mL streptomycin at 37°C in 5% CO_2_ (49).

***In vitro* toxicity assay.** The cytotoxicity of TP4-3 was evaluated by an alamarBlue assay. HaCaT, HK-2, and RAW 264.7 macrophage cells were seeded at densities of 1 × 10^4^ cells/well in 96-well cell plates and treated with the indicated concentrations of TP4 and TP4-3 for 24 h (0.78 to 100 μM). Cells treated with 0.1% Triton X-100 and culture medium served as negative and positive-control groups, respectively. After incubation, the cells were washed and incubated for 2 h at 37°C with 90 μL Dulbecco’s modified Eagle medium (DMEM) containing 10 μL alamarBlue reagent. Fluorescence was recorded using a Spectra Max i3 Multi-Mode detection platform (Molecular Devices, San Jose, CA) to measure cell viability (50, 51).

### Bacterial killing kinetics.

Mid-log-phase A. baumannii (5 × 10^5^ CFU/mL) bacterial cells were treated with the test candidates at appropriate bactericidal concentrations, followed by incubation at 37°C. The treated bacterial cells were plated in MHA to determine the bacterial killing rate as a function of time (52).

### PI and NPN membrane permeabilization assays.

Mid-log-phase bacterial cells (A. baumannii) were resuspended in 10 mM PBS (pH 7.4) to a density of 10^8^ CFU/mL. The cell suspension was supplemented with 25 mM glucose followed by 15 min incubation at 37°C. After the incubation, *N*-phenyl-1-naphthylamine dye was added to a final concentration of 10 μM. Next, 90 μL of bacterial cells was transferred to a 96-well black plate, and fluorescence was recorded for 40 min (excitation/emission: 350/420 nm). Bacterial cells were then treated with 10 μL of test compound at the MIC (25 μM). After this treatment, fluorescence was recorded for an additional 20 min (53). Data are presented as fluorescence values as a function of time. Permeabilization of blank- and test compound-treated cells was compared. For the PI membrane permeabilization assay, 10^8^ bacterial cells (A. baumannii) were resuspended in MHB containing 20 μg/mL PI. Next, 90 μL of the bacterial suspension was treated with 10 μL of the test compound at its MIC (25 μM) in a 96-well black plate. Fluorescence was read (excitation/emission: 584/620 nm). Fluorescence values of blank- and compound-treated cells were graphed as a function of time (54, 55).

### DiBAC4(3) membrane depolarization assay.

Mid-log-phase A. baumannii bacterial cells were suspended in 10 mM PBS (pH 7.4) at a density of 10^8^ CFU/mL. The cell suspension was then supplemented with 25 mM glucose, followed by 15 min incubation at 37°C. Then, the cell suspension was mixed with 500 nM DiBAC4(3) and incubated for 50 min. After incubation, 90 μL of the cell suspension was transferred to a 96-well plate, and fluorescence was recorded (excitation/emission: 480/520 nm) until the signal stabilized (~40 min). Next, 10 μL of the test compound at its MIC (25 μM) was added to each well, followed by fluorescence recording for an additional 40 min. Data for blank- and compound-treated groups were compared by plotting fluorescence as function of time (56).

### Serial passage assay.

The experimental procedure was performed according to the MIC protocol for A. baumannii described above. Subsequently, bacterial samples from sub-MIC wells were passaged, and the MIC was estimated again. This process was repeated for 15 cycles, and the change in MIC over 10 passages was reported (23).

### Antibiofilm assay.

**(i) Biofilm inhibition assay.** Fifty μL of 2 × 10^6^ CFU/mL A. baumannii bacterial suspension was added to a 96-well plate. Then, 50 μL of peptide/drug (various concentrations) was added, and the plate was incubated for 24 h. The residual solution was removed, and wells were washed, followed by treatment with 100% methanol for 15 min at room temperature. The methanol was then removed, and the wells were allowed to dry at room temperature. Next, the wells were incubated with 100 μL of 0.02% crystal violet for 30 min. After the incubation, the wells were washed with distilled water and dried for an appropriate length of time. Finally, 100 μL of 33% acetic acid was added to the wells for 1 h, followed by reading the absorbance at 595 nm (55).

**(ii) Biofilm rupture assay.** Fifty μL of A. baumannii bacterial strains suspension cultures (2 × 10^6^ CFU/mL) were plated and incubated in a shaking incubator for 24 h. Then, the wells were washed with PBS and treated with 100 μL of peptide/drug at various concentrations. The plates were incubated for 24 h and then washed with PBS. The wells were treated with 100% methanol for 15 min, and the residual solution was washed followed by drying at room temperature. After the wells were dry, 100 μL of 0.02% crystal violet solution was added, followed by incubation for 30 min at room temperature. The wells were then washed with water and dried for an appropriate length of time. Once the wells were dry, 100 μL of 33% acetic acid was added to the wells and they were incubated at room temperature for 1 h. Absorbance was recorded at 595 nm (55).

### Fractional inhibitory concentration index.

The FICI was measured using a standard checkerboard microdilution assay (57). Two-fold dilutions of antibiotics were made across the *x* axis, and two-fold peptide dilutions were made across the *y* axis. Next, bacteria were added at 5 × 10^5^ CFU/well (A. baumannii), followed by incubation at 37 ± 2°C for 16 to 20 h. FIC indices were calculated according to the following equations: FICI = FIC_1_ + FIC_2_; FIC_1_ = (MIC_12_ combined)/MIC_1_; FIC_2_ = (MIC_12_ combined)/MIC_2_. The combined efficacy was estimated as follows: FICI ≤ 0.5, synergistic; 0.5 to 1, additive; 1 to 2, indifferent; ≥2, antagonistic.

### Scanning electron microscopy and transmission electron microscopy analysis.

A cell suspension of 10^8^ A. baumannii was treated with peptide at its MIC and incubated overnight at 37 ± 2°C. The treated suspension was washed twice with 10 mM PBS (pH 7.4) followed by treatment with 4% glutaraldehyde. Next, the suspension was incubated for 30 min at 4°C. Following the incubation, the treated suspension was centrifuged (5,000 × *g*, 10 min), and the pellet was placed on a glass slide and allowed to attach for 30 min. The glass slide was then subjected to gradient ethanol washing from 0% to 100% ethanol. Finally, the glass slides were dried and coated with gold for imaging (55). For TEM, glutaraldehyde samples were cryoimmobilized and subjected to TEM imaging (58, 59).

### *In vivo* toxicity.

The *in vivo* toxicity of TP4-3 was assessed in female C57BL/6 mice (BioLasco, Ilan, Taiwan) upon intravenous administration of TP4-3 (9, 45, and 60 mg/kg). Mice treated with saline or meropenem (10 mg/kg) served as negative- and positive-control groups, respectively. All animals were monitored for 7 days. The number of dead animals and animal body weights were measured every 24 h. Seven days after the drug administration, blood samples were collected, and the animals were euthanized. ALT (GPT), AST (GOT), CRE, and BUN were analyzed using a dry-chemistry analyzer (Fujifilm DRI-CHEM 4000i) and commercially available kits (Fujifilm Corp., Tokyo, Japan) (23, 54). All animal experiments were approved by the institutional ethical committee (IACUC, Academia Sinica; ID no. 2021-12-1577 and 2021-12-1771).

### *In vivo* efficacy of TP4-3 in CLP-induced polymicrobial sepsis model.

**(i) CLP surgery.** Cecal ligation and puncture surgery was performed to induce polymicrobial sepsis according to a previously described method (37). Female C57BL/6 mice at 6 to 8 weeks of age (weight = 18 to 22 g) were anesthetized by intraperitoneal administration of Zoletil (50 mg/kg). Then, a 1-cm incision was made at the abdomen to expose the cecum. The CLP procedure was performed with a 23-G needle. Thirty minutes after the surgery, animals (*n* = 8) were treated with saline (C+S), TP4-3 (9 mg/kg, C + T), meropenem (10 mg/kg, C + M), TP4 (4.5 mg/kg, C + TP4) or combined treatment of meropenem (5 mg/kg) and TP4-3 (C + T + M) via intraperitoneal injection. A sham group was subjected to abdomen incision without CLP and treated with saline by intraperitoneal injection. After the treatments, percent survival was used as a measure of test-candidate efficacy (37).

**(ii) Estimation of bacterial load, cytokine expression, and toxicity profile after CLP and treatment.** At 24 h after the CLP surgery, mice (*n* = 6) were anesthetized by intraperitoneal administration of 50 mg/kg Zoletil. Blood samples were taken in collection tubes. Peritoneal fluid was collected in a sterilized 1.5-mL centrifuge tube. Organs, including the liver, lung, spleen, and kidney, were harvested, weighed, and placed in 1-mL sterile PBS for homogenization with a tissue lyzer (Tissuelyzer II, Qiagen, Dusseldorf, Germany). The homogenates, blood, and peritoneal fluid were diluted serially and plated on Mueller-Hinton broth agar plates. The plates were incubated at 37°C overnight. Each tissue was evaluated by measuring CFU/g (37).

To evaluate kidney and liver function, blood samples (*n* = 5 per group) were tested for ALT (GPT), AST (GOT), CRE, and BUN using a dry-chemistry analyzer as described above. Mouse serum was extracted to assess (*n* = 5 per group) expression of cytokines (Thermo Fisher Scientific, Waltham, MA) as a measure of inflammatory response (37).
