# Supplementary material for: Rational Design of Stapled Antimicrobial Peptides to Enhance Stability and In Vivo Potency against Polymicrobial Sepsis
Source: Microbiol Spectr. 2023 Mar 6;11(2):e03853-22. doi: 10.1128/spectrum.03853-22 (PMC10101059; doi:10.1128/spectrum.03853-22)
Supplement: Supplemental file 1 — Supplemental material. Download spectrum.03853-22-s0001.pdf, PDF file, 1.9 MB [file spectrum.03853-22-s0001.pdf]

Supplemental File

Rational design of stapled antimicrobial peptides to enhance stability and *invivo* potency against polymicrobial sepsis

Stapled peptides display better *in vivo* activity

Jih-Chao Yeh [a ‡], Prakash Kishore Hazam [a ‡], Chu-Yi Hsieh [a], Po- Hsien Hsu [b], Wen-Chun Lin [a], Yun-Ru Chen [c], Chao-Chin Li [d], Jyh-Yih Chen [a,e*]

a. Marine Research Station, Institute of Cellular and Organismic Biology, Academia Sinica, 23-10 Dahuen Rd., Jiaushi, Ilan 262, Taiwan

b. Institute of Fisheries Science, National Taiwan University, 1 Roosevelt Road, Sec. 4, Taipei 106, Taiwan

c. Academia Sinica Protein Clinic, Institute of Biological Chemistry, Academia Sinica, 128, Academia Road, Section 2, Nankang District, Taipei 115, Taiwan

d. Institute of Cellular and Organismic Biology, Academia Sinica, Nankang, Taipei 115, Taiwan

e. The iEGG and Animal Biotechnology Center and the Rong Hsing Research Center for Translational Medicine, National Chung Hsing University, Taichung 402, Taiwan

‡ These authors contributed equally as joint first authors.

*Corresponding Author: Jyh-Yih Chen
Email id: zoocjy@gate.sinica.edu.tw

Table of contents

S. no	Content	Page no
1	Fig. S1	2
2	Fig. S2	2
3	Fig. S3 a, b	3-4
4	Fig. S4 a, b	5-6
5	Fig. S 5 a, b	7-8
6	Fig. S6 a, b	9-10
7	Fig. S7	11
8	Table. S1	11
9	Fig. S8	12
10	Table. S2	13
11	Fig. S9	14
12	Fig. S10	15-16

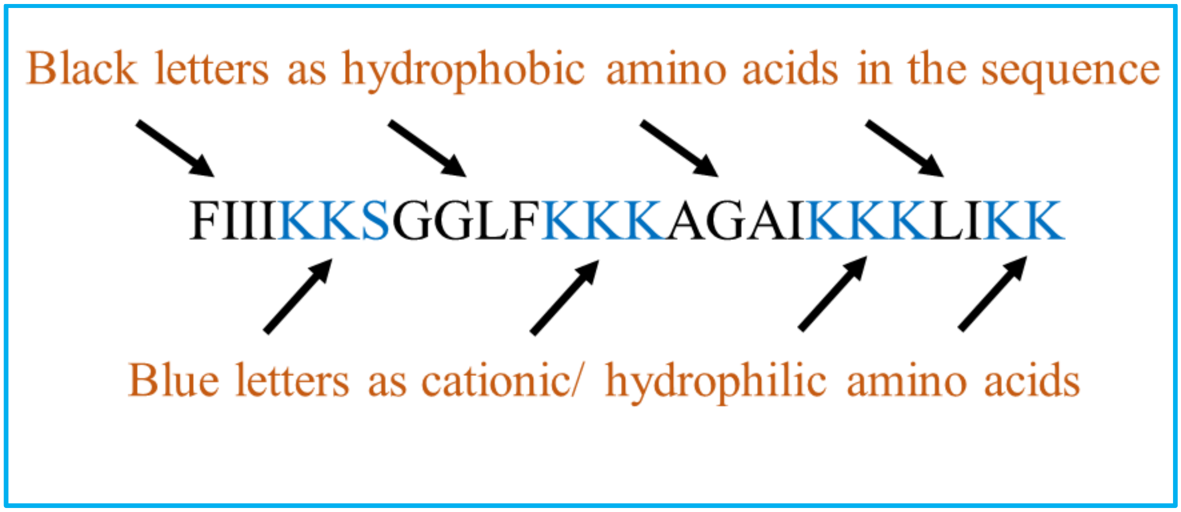

Fig. S1: Schematic representation of the peptide sequence after replacing arginine and histidine with lysine. The peptide was subdivided into four hydrophobic and three cationic/ hydrophilic segments. Letters in black indicate hydrophobic amino acids, whereas blue letters show the cationic/ hydrophilic amino acids in the peptide sequence.

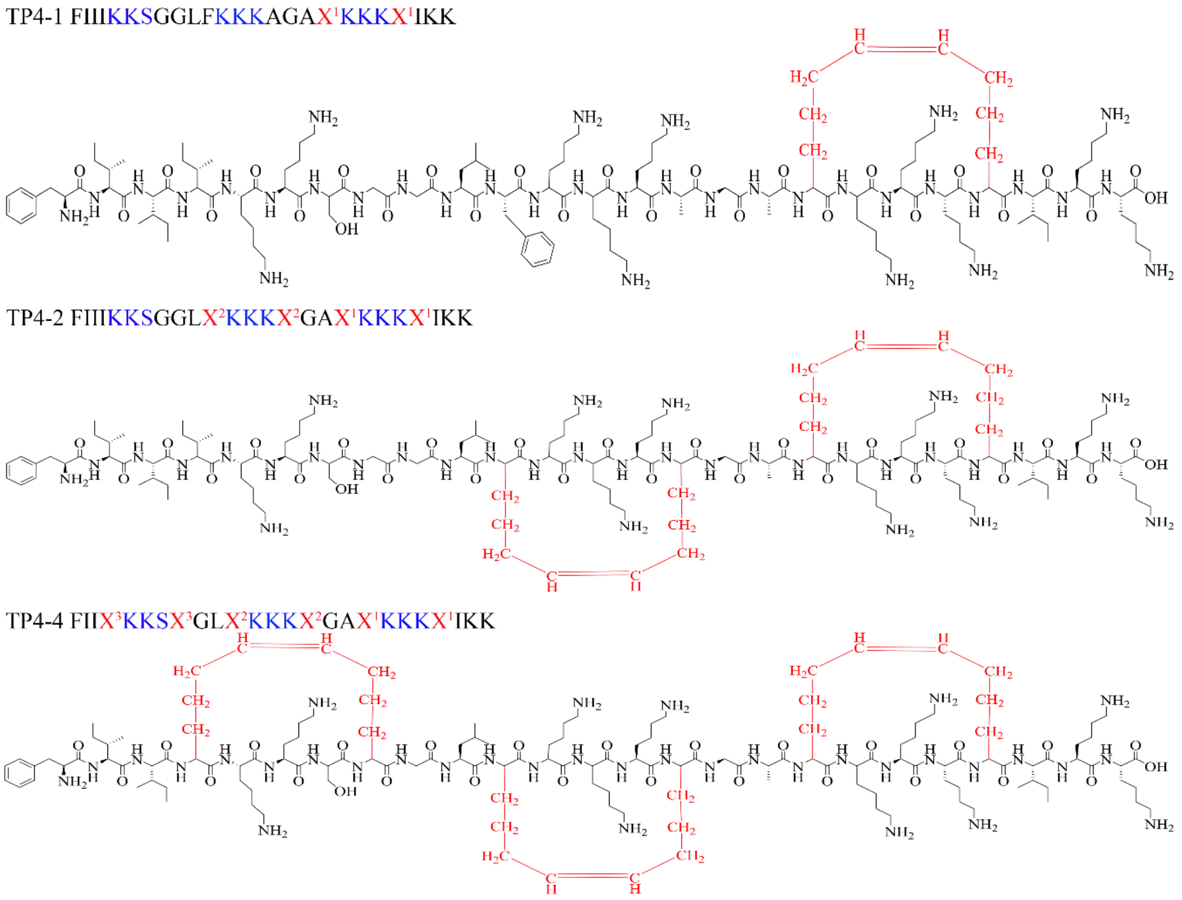

Fig. S2: Schematic representations of the chemical structures of TP4-1, TP4-2, and TP4-4. The X letters (red, blue) show the positions of stapling, with the same numbers indicating the stapling

pairs within the given sequence.

HPLC REPORT

Product Name	:A1 FK-23
Lot No	:P200123-HS778290
Column	:Gemini-NX 5μ C18 110A, 4.6*250mm
Solvent A	:0.1%Trifluoroacetic in 100% Acetonitrile
Solvent B	:0.1%Trifluoroacetic in 100% Water
Gradient	:

	A	B
0.01min	10%	90%
25min	70%	30%
25.01min	100%	0%
30min	Stop	

Flow rate	:1.0ml/min
Wavelength	:220nm
Volume	:20ul

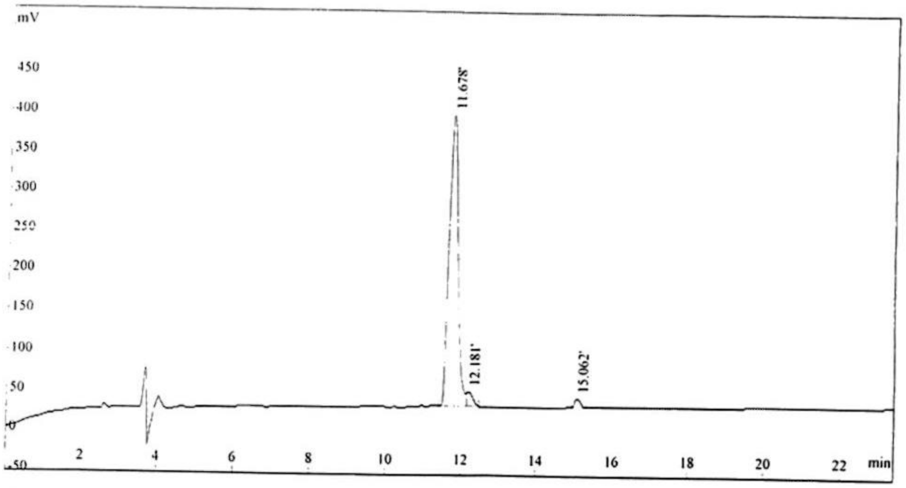

Rank	Time	Conc.	Area	Height
1	11.678	95.2875	5884083	364396
2	12.181	2.9644	183055	17396
3	15.062	1.7481	107946	10906
Total		100	6175084	392698

Fig. S3a: HPLC chromatogram showing percent purity of the synthesized peptide TP4-1.

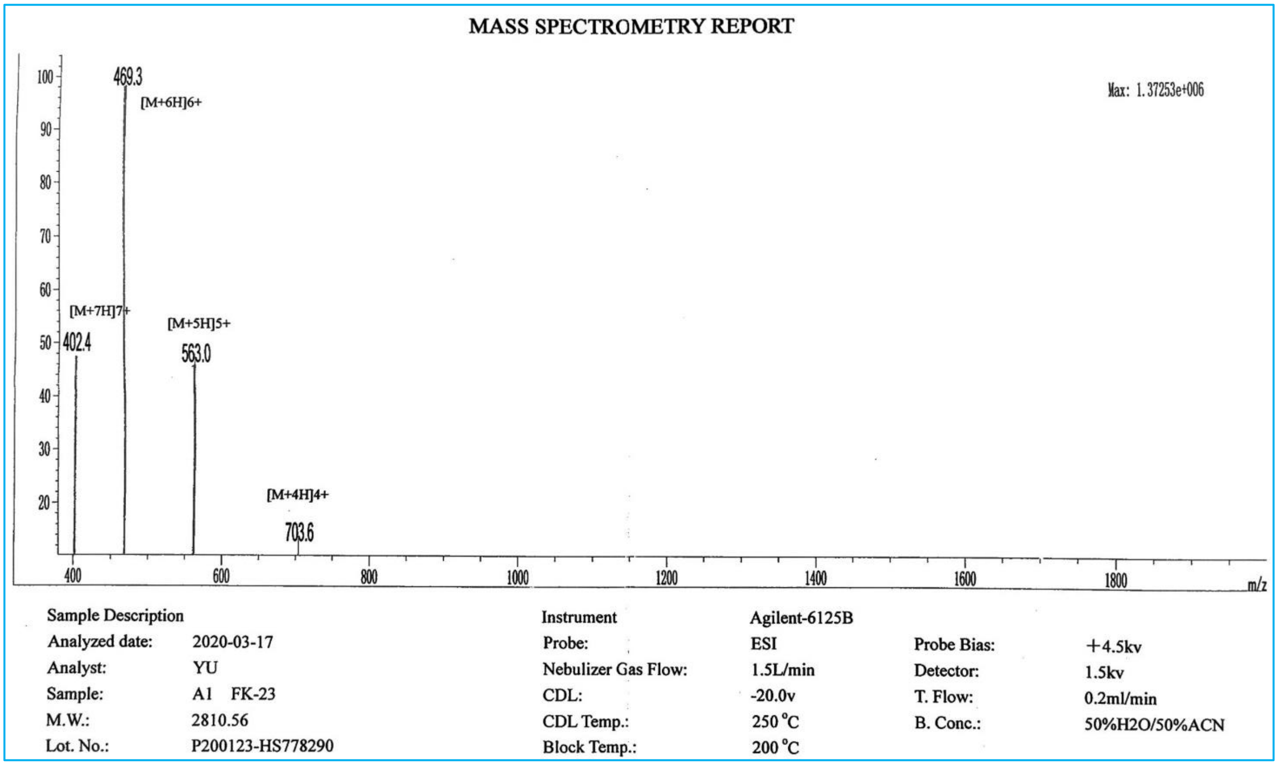

Fig. S3b: Mass spectrometry-based characterization of peptide TP4-1.

HPLC REPORT

Product Name	:A2 FK-22
Lot No	:P200123-HS778291
Column	:Gemini-NX 5μ C18 110A, 4.6*250mm
Solvent A	:0.1%Trifluoroacetic in 100% Acetonitrile
Solvent B	:0.1%Trifluoroacetic in 100% Water

Gradient :

	A	B
0.01min	15%	85%
25min	75%	25%
25.01min	100%	0%
30min	Stop	

Flow rate	:1.0ml/min
Wavelength	:220nm
Volume	:20ul

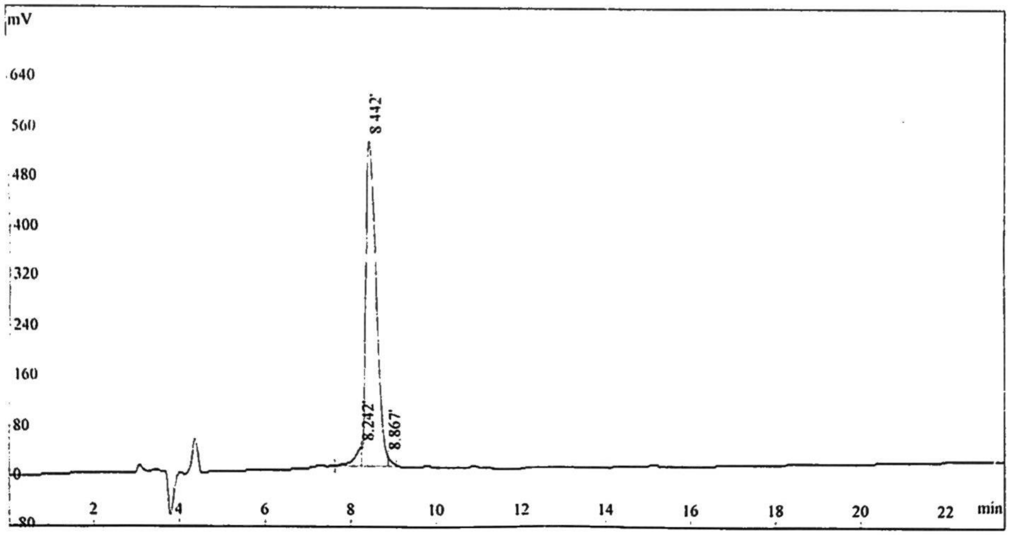

Rank	Time	Conc.	Area	Height
1	8.242	3.6928	321761	28820
2	8.442	95.6186	8331367	520204
3	8.867	0.6886	59994	12552
Total		100	8713122	561576

Fig. S4a: HPLC chromatogram showing percent purity of the synthesized peptide TP4-2.

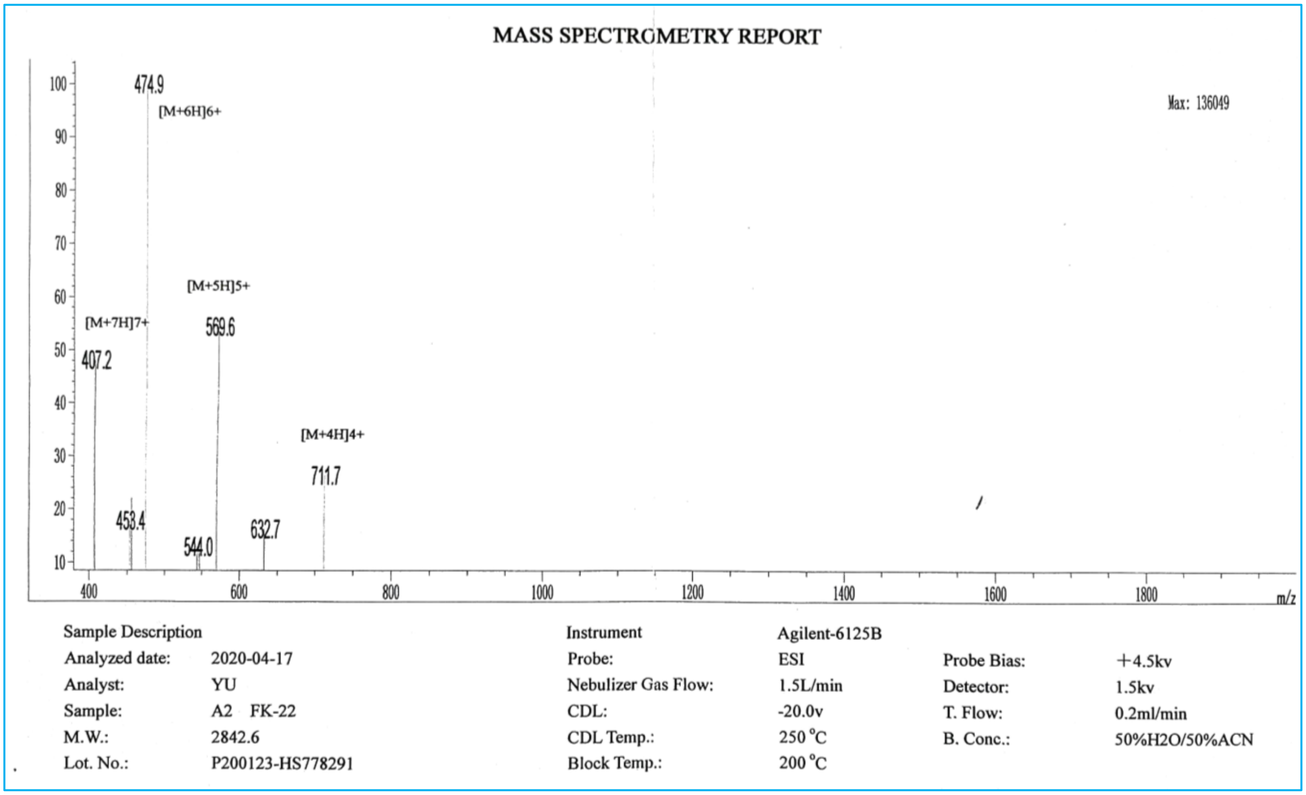

Fig. S4b: Mass spectrometry-based characterization of TP4-2 peptide.

HPLC REPORT

Product Name	:TP4-3 FK-21
Lot No	:P200810-HS823976
Column	:Gemini-NX 5μ C18 110A, 4.6*250mm
Solvent A	:0.1%Trifluoroacetic in 100% Acetonitrile
Solvent B	:0.1%Trifluoroacetic in 100% Water

Gradient	:	A	B
	0.01min	10%	90%
	25min	70%	30%
	25.01min	100%	0%
	30min	Stop	

Flow rate	:1.0ml/min
Wavelength	:220nm
Volume	:20ul

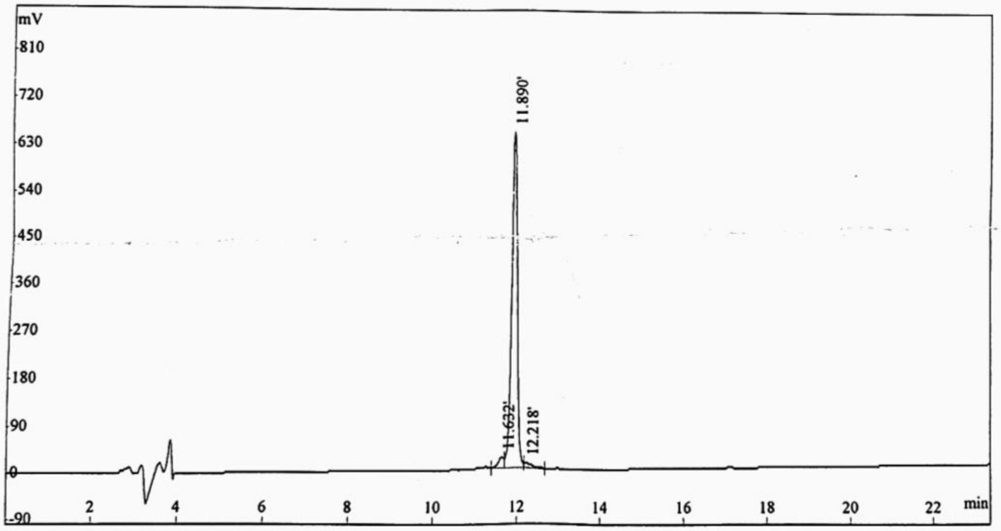

Rank	Time	Conc.	Area	Height
1	11.632	2.9184	175742	20139
2	11.890	95.0826	5725720	644387
3	12.218	1.9990	120374	8796
Total		100	6021836	673322

Fig. S5a: HPLC chromatogram showing percent purity of the synthesized peptide TP4-3.

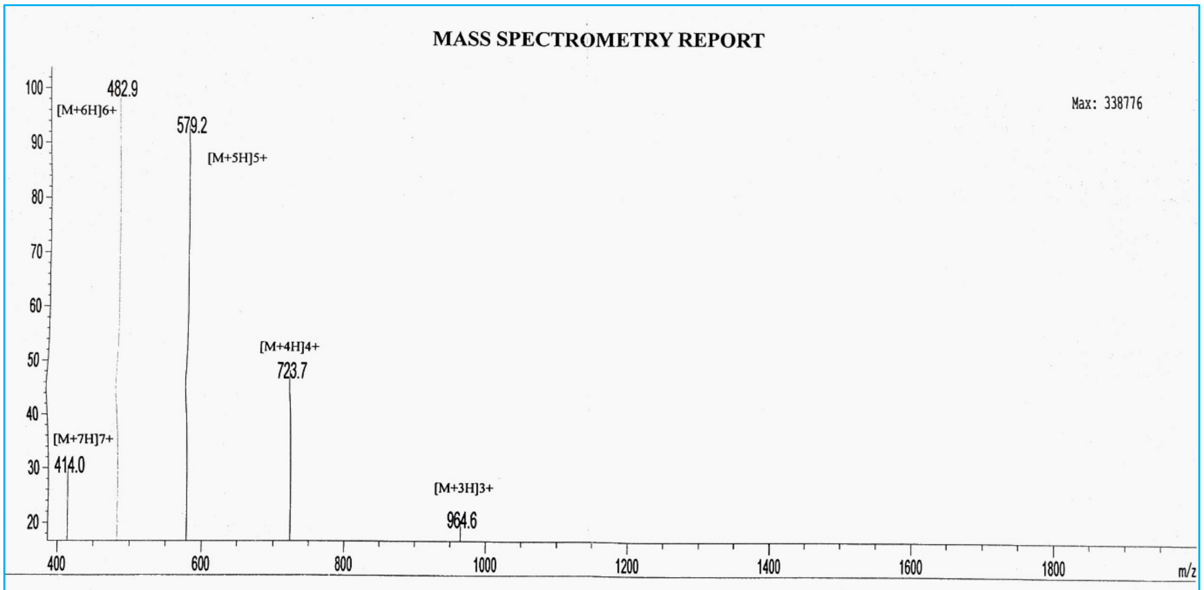

Sample Description		Instrument	Agilent-6125B		
Analyzed date:	2020-11-13	Probe:	ESI	Probe Bias:	+4.5kv
Analyst:	YU	Nebulizer Gas Flow:	1.5L/min	Detector:	1.5kv
Sample:	TP4-3 FK-21	CDL:	-20.0v	T. Flow:	0.2ml/min
M.W.:	2890.65	CDL Temp.:	250 °C	B. Conc.:	50%H2O/50%ACN
Lot. No.:	P200810-HS823976	Block Temp.:	200 °C		

Fig. S5b: Mass spectrometry-based characterization of TP4-3 peptide.

HPLC REPORT

Product Name	:A5 FK-21
Lot No	:P200123-HS778294
Column	:Gemini-NX 5 μ C18 110A, 4.6*250mm
Solvent A	:0.1%Trifluoroacetic in 100% Acetonitrile
Solvent B	:0.1%Trifluoroacetic in 100% Water

Gradient :

	A	B
0.01min	20%	80%
25min	80%	20%
25.01min	100%	0%
30min	Stop	

Flow rate	:1.0ml/min
Wavelength	:220nm
Volume	:20ul

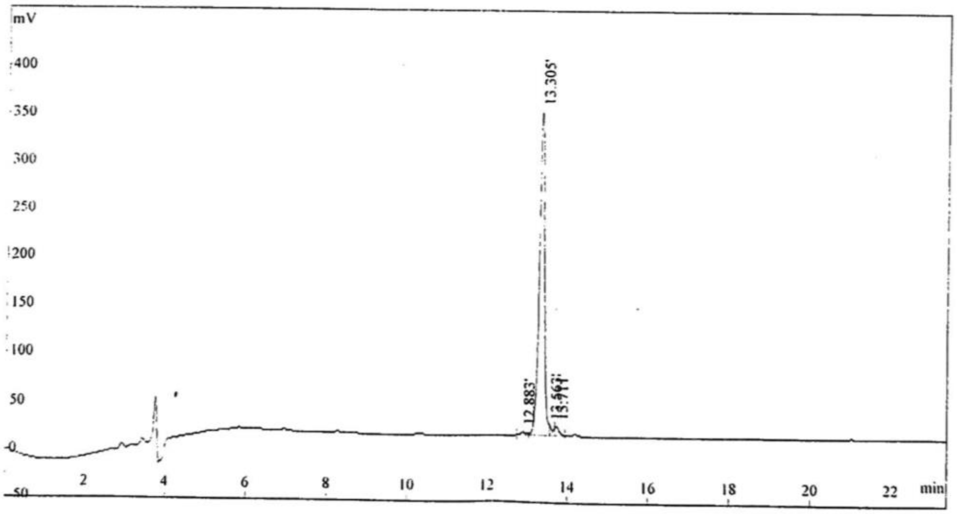

Rank	Time	Conc.	Area	Height
1	12.883	0.9532	28719	2873
2	13.305	95.5710	2879476	335880
3	13.563	1.4271	42996	9093
4	13.711	2.0487	61726	9510
Total		100	3012917	357356

Fig. S6a: HPLC chromatogram showing percent purity of the synthesized peptide TP4-4.

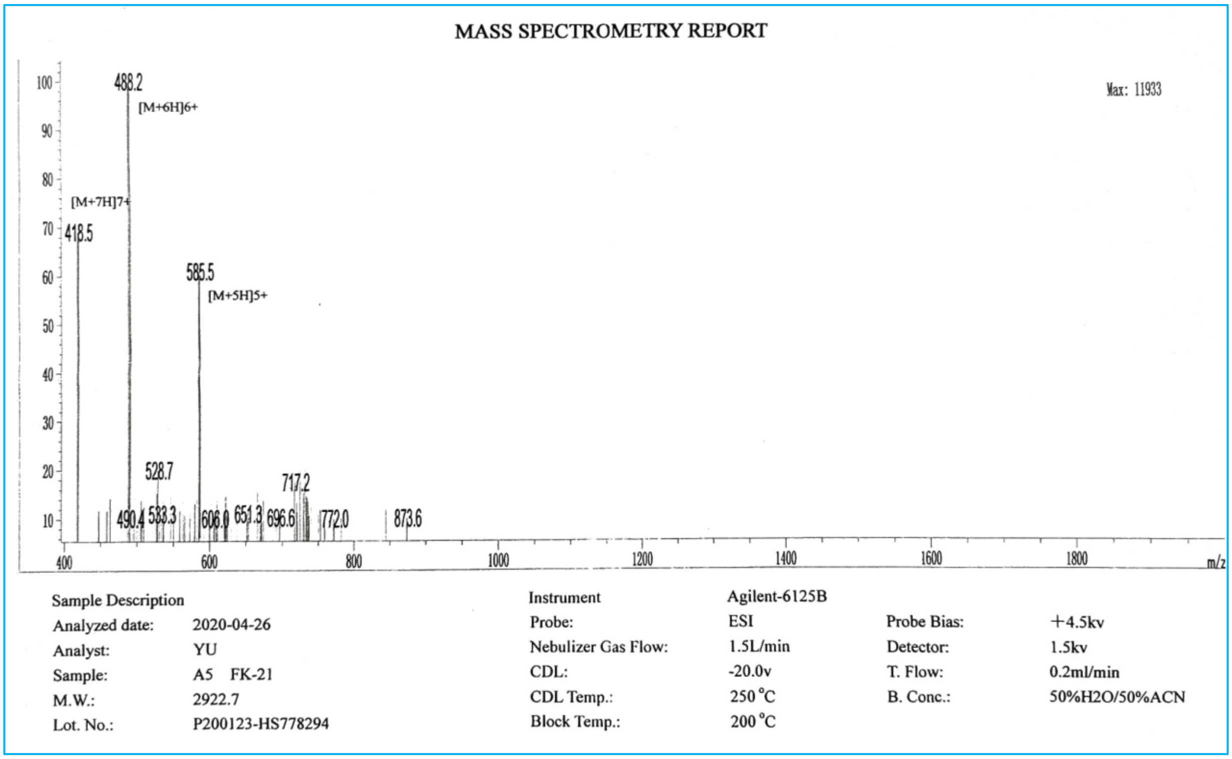

Fig. S6b: Mass spectrometry-based characterization of TP4-4 peptide.

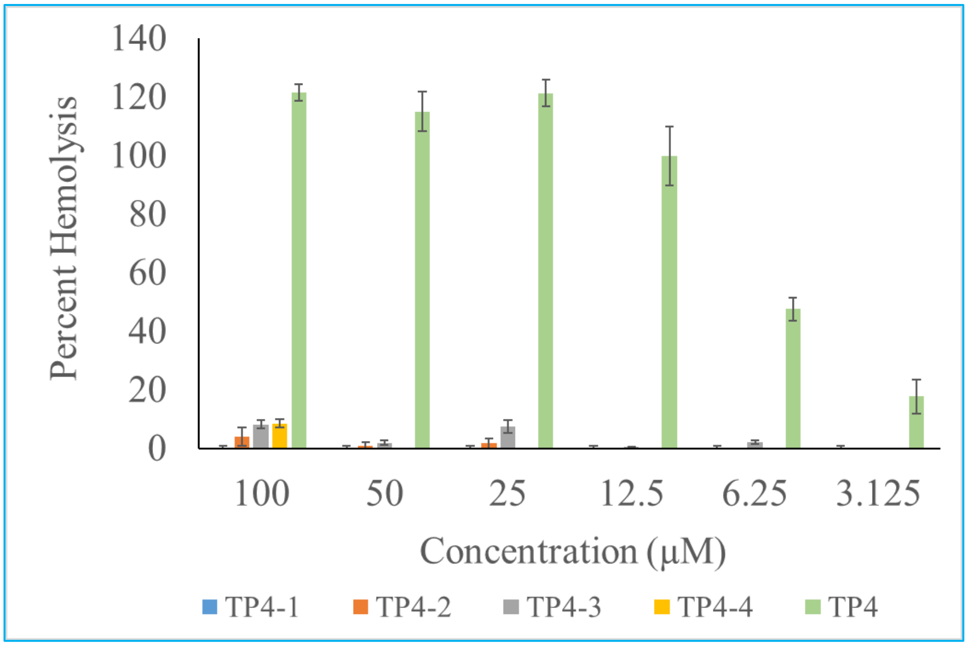

Fig. S7: Hemolysis of peptides tested with human RBCs. The concentrations are expressed in µM.

Table S1: *A. baumannii* MDR strain resistance to different clinical antibiotics.

S. no	Bacteria strain number	Multiplex PCR	OXA51-like	OXA58-like	Ami	Amp-sul	Cef	Cefta z	Cipro	Col	Dor	Gen	Imp	Mer	Pip-Taz	Sul	Tig
1	921	Ab	+	+	>2048	64/32	NA	128	128	1	NA	>1024	32	64		16	2
2	1019	Ab	+	+	>2048	64/32	NA	256	64	1	NA	>1024	32	16	1024/4	32	1
3	1033	Ab	+	+	>2048	NA	32	256	64	1	32	>1024	32	64	256/4	16	2
4	1607	Ab	+	+	>32	NA	NA	NA	>2	1	NA	>8	32	32	>64	16	NA
5	1702	Ab	+	+	>2048	NA	32	512	256	1	NA	>1024	32	16	2048/4	8	0.5
6	2962	Ab	+	+	>128	NA	>32	>32	64	2	128	>32	16	16	NA	NA	NA
7	2982	Ab	+	+	>2048	128/64	128	128	128	4	NA	>4096	64	128	4096/4	NA	NA
8	2997	Ab	+	+	>2048	128/64	128	512	>8	4	32	>4096	64	64	1024/4	NA	NA
9	2998	Ab	+	+	>2048	128/64	256	128	128	4	NA	>4096	64	128	4096/4	32	2
10	3618	Ab	+	+	>2048	128/64	64	512	256	4	64	>1024	32	64	1024/4	NA	NA

Ami = Amikacin, Amp-sul = Ampicillin-sulbactam, Cef = Cefepime, Cefz = Ceftazidime, Cipro = Ciprofloxacin, Col = Colistin, Dor = Doripenem,Gen = Gentamicin, Imp = Imipenem, Mer = Meropenem, Pip-Taz = Piperacillin-Tazobactam, Sul = Sulbactam, Tig = Tigecycline, NA = Data not available.

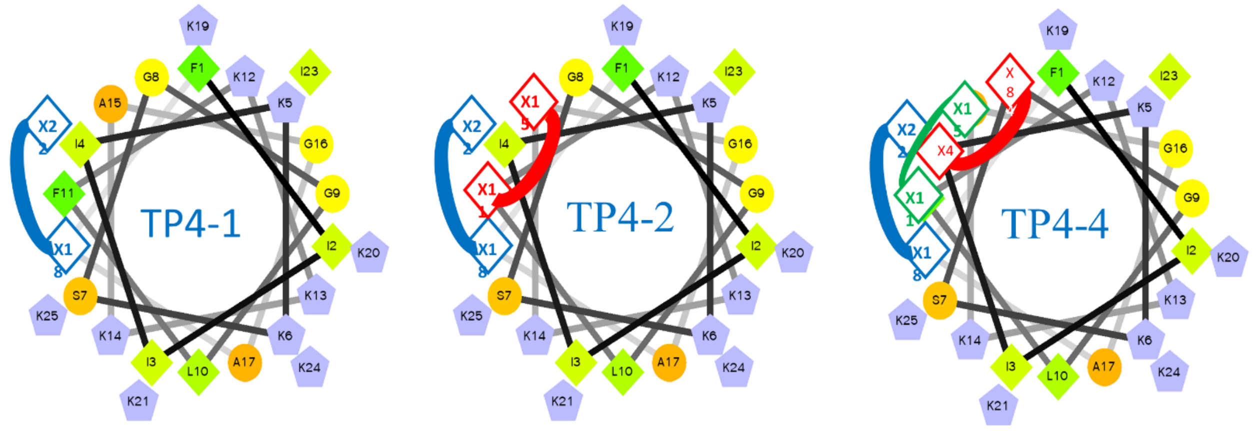

Fig. S8: Schematic representations (helical wheels) of peptides TP4-1, TP4-2 and TP4-4. The X letters (red, blue, green) show the positions of stapling, with the same numbers indicating stapling pairs within the given sequence.

Table S2: FIC indices of TP4-3 with meropenem (M) or doxycycline (D) against different MDR *A. baumannii*, an NDM1 *K. pneumonia*, and selected wild-type bacterial species. The values are expressed in µM. The experiments were repeated twice, with each sample tested in triplicate.

**Bacterial species**		**Single drug treatment MIC**			**TP4-3 and meropenem combined MIC**		**FICI of TP4-3 + M**	**TP4-3 and meropenem combined MIC**		**FICI of TP4-3 + D**
		**TP4-3**	**M**	**D**	**TP4-3**	**M**		**TP4-3**	**D**	
MDR *A. baumannii*	14B001	3.13	800	50	0.09	400	0.53	3.13	50	1
	2088	6.25	400	100	3.13	100	0.75	3.13	12.5	0.63
	921	6.25	400	200	0.78	100	0.38	3.13	25	0.56
	1019	12.5	50	100	3.13	6.25	0.38	3.13	12.5	0.31
	1033	12.5	200	50	3.13	50	0.5	3.13	12.5	0.5
	1607	6.25	100	100	3.13	12.5	0.63	3.13	25	0.75
	1702	6.25	25-50	50	3.13	12.5	0.75	3.13	12.5	0.75
	2962	6.25	100	100	3.13	25	0.75	3.13	25	0.75
	2982	6.25	400	50	3.13	200	1	3.13	25	1
	2997	6.25	200	50	3.13	12.5	0.56	3.13	25	1
	2998	6.25	400	50	3.13	50	0.63	3.13	12.5	0.75
	3618	12.5	200	100	3.13	25	0.38	3.13	25	0.5
NDM1*K. pneumoniae*		25	400	25	6.25	200	0.75	6.25	3.13	0.38
E. aerogenes		25	0.39	3.13	0.78	0.097	0.28	3.13	0.78	0.25
E. coli		25	3.13	50	0.78	0.097	0.28	6.25	0.78	0.5
S enterica		12.5	6.25	6.25	0.39	0.19	0.28	3.13	1.56	0.5
P. aeruginosae		25	0.39	6.25	0.78	0.78	0.28	6.25	12.5	0.5

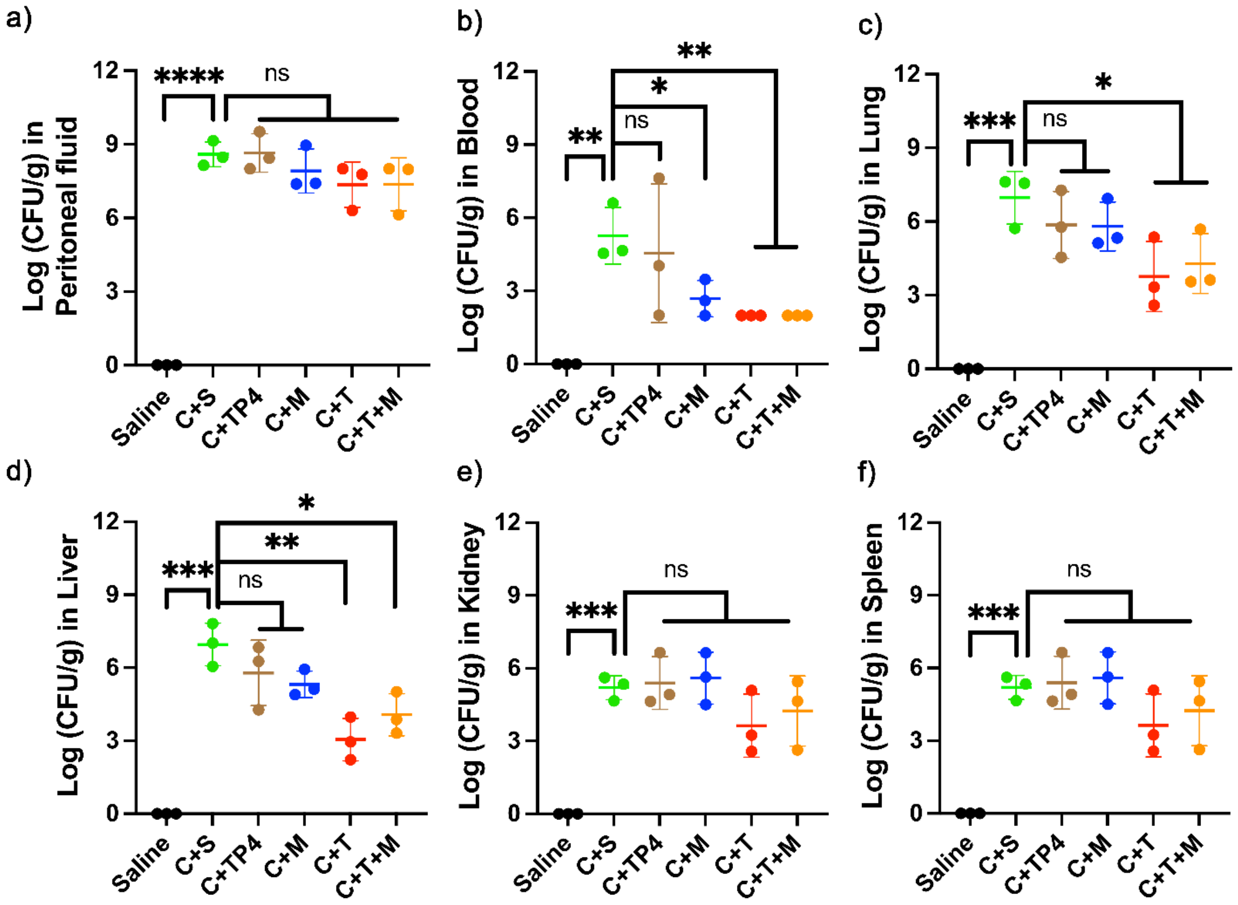

Fig. S9: Protective effects of TP4-3 and combination of TP4-3 with meropenem on bacterial burden 72 h after cecal ligation puncture (CLP). Thirty minutes after surgery mice were treated intraperitoneal injection of saline (C+S), 9 mg/kg TP4 (C+TP4), 10 mg/kg Meropenem (C+M), 9 mg/kg TP4-3 (C+T), and 9 mg/kg TP4-3 with 5 mg/kg Meropenem (C+T+M). Seventy-two hours after treatment, a) peritoneal fluid, b) blood, c) lung, d) liver, e) kidney, and f) spleen were collected and plated for analysis (n = 3). *p < 0.05, **p < 0.01, ***p < 0.001, ****p < 0.0001; ns: no statistical significance.

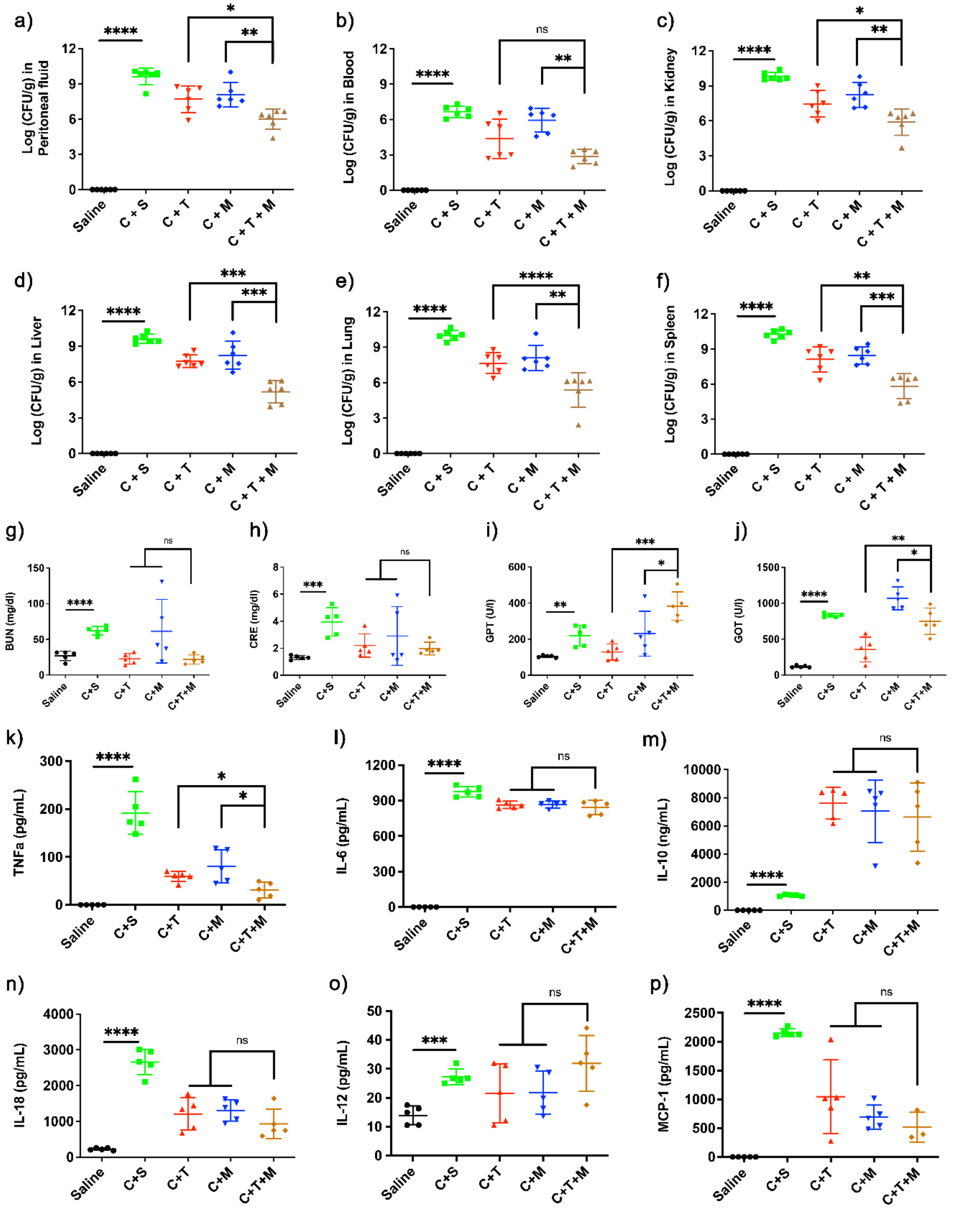

Fig. S10. Protective effects of combined treatment of peptide TP4-3 with meropenem on bacterial burden, kidney function, liver function, and cytokines induced by cecal ligation and puncture (CLP). CLP surgery was performed on female C57BL/6 mice. Thirty minutes later, intraperitoneal administration of saline (C+S), TP4-3 (9 mg/kg, C+T), Meropenem (10 mg/kg, C+M), and TP4-3 + Meropenem (9mg/kg TP4-3 and 5 mg/kg meropenem, C+T+M) (n = 8) was performed. Twenty-

four hours after surgery, the peritoneal fluid (a), blood (b), lung (c), liver (d), kidney (e), and spleen (f) were collected and plated for analysis (n = 6). BUN concentration (g); creatinine level (h); GPT activity (i), and GOT activity (j) were also measured from serum to assess kidney and liver functions (n = 5). TNFα (k), IL-6 (l), IL-18 (m), IL-10 (n), IL-12 (o), and MCP-1 (p) were measured by ELISA from serum collected at 24 h (n = 5). *$p < 0.05$, **$p < 0.01$, ***$p < 0.001$, and ****$p < 0.0001$, ns: no statistical significance.
